# Molecular correlates of vaccine-induced protection against typhoid fever

**DOI:** 10.1172/JCI169676

**Published:** 2023-08-15

**Authors:** Henderson Zhu, Irina Chelysheva, Deborah L. Cross, Luke Blackwell, Celina Jin, Malick M. Gibani, Elizabeth Jones, Jennifer Hill, Johannes Trück, Dominic F. Kelly, Christoph J. Blohmke, Andrew J. Pollard, Daniel O’Connor

**Affiliations:** 1 Oxford Vaccine Group, Department of Paediatrics, University of Oxford, Oxford, United Kingdom.; 2 NIHR Oxford Biomedical Research Centre and Oxford University Hospitals NHS Foundation Trust, Oxford, United Kingdom.; 3 Department of Infectious Disease, Imperial College London, St Mary’s Campus, London, United Kingdom.; 4 Division of Immunology, University Children’s Hospital Zurich, Zurich, Switzerland.

**Keywords:** Vaccines, Bacterial infections, Bacterial vaccines, Bioinformatics

## Abstract

**BACKGROUND:**

Typhoid fever is caused by the Gram-negative bacterium *Salmonella enterica* serovar Typhi and poses a substantial public health burden worldwide. Vaccines have been developed based on the surface Vi-capsular polysaccharide of *S.* Typhi; these include a plain-polysaccharide-based vaccine, ViPS, and a glycoconjugate vaccine, ViTT. To understand immune responses to these vaccines and their vaccine-induced immunological protection, molecular signatures were analyzed using bioinformatic approaches.

**METHODS:**

Bulk RNA-Seq data were generated from blood samples obtained from adult human volunteers enrolled in a vaccine trial, who were then challenged with *S.* Typhi in a controlled human infection model (CHIM). These data were used to conduct differential gene expression analyses, gene set and modular analyses, B cell repertoire analyses, and time-course analyses at various post-vaccination and post-challenge time points between participants receiving ViTT, ViPS, or a control meningococcal vaccine.

**RESULTS:**

Transcriptomic responses revealed strong differential molecular signatures between the 2 typhoid vaccines, mostly driven by the upregulation in humoral immune signatures, including selective usage of immunoglobulin heavy chain variable region (*IGHV*) genes and more polarized clonal expansions. We describe several molecular correlates of protection against *S.* Typhi infection, including clusters of B cell receptor (BCR) clonotypes associated with protection, with known binders of Vi-polysaccharide among these.

**CONCLUSION:**

The study reports a series of contemporary analyses that reveal the transcriptomic signatures after vaccination and infectious challenge, while identifying molecular correlates of protection that may inform future vaccine design and assessment.

**TRIAL REGISTRATION:**

ClinicalTrials.gov NCT02324751.

## Introduction

Typhoid fever is an urgent public health problem in resource-limited regions of the world, causing approximately 10.9 million cases and 100,000 deaths annually (1). Increasing antimicrobial resistance (AMR) presents a challenge to the treatment of typhoid fever. Preventative measures, including water, sanitation, and hygiene (WASH) interventions, coupled with the deployment of effective vaccines, were implemented to reduce the burden of the disease.

**Figure 1:**
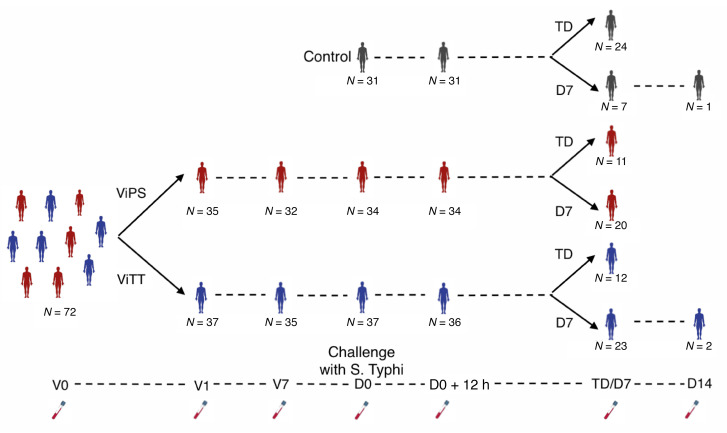
Study design. Graphical overview of the study. Participants were classified as TD if they had a fever of 38°C or higher for 12 or more hours and/or had *S*. Typhi cultured from blood in the 14 days following challenge. Whole blood samples were collected at the following selected time points: for ViTT and ViPS recipients, on V0, V1, V7, D0, and D0+12h; for control group participants, on D0 and D0+12h; for nTD participants, on D7 and D14; and for TD participants, on the day of typhoid diagnosis (TD).

Typhoid conjugate vaccines have emerged as an effective method of controlling typhoid fever. We have previously described the efficacy of Vi–polysaccharide–tetanus toxoid glycoconjugate vaccine (ViTT, also known as ViTCV) in a controlled human infection model (CHIM) study (Figure 1 and Table 1), in which ViTT was at least 50% efficacious at preventing culture confirmed disease. The efficacy of ViTT has been confirmed in large phase III field trials, where 80% efficacy has been observed in children (2–4). In comparison, a licensed plain Vi-polysaccharide vaccine (ViPS) showed 60% efficacy in children (5). There is an incomplete understanding of vaccine-induced immunological protection against typhoid fever (6, 7). CHIM studies allow a more detailed characterization of the host response to vaccination and infection than is typically possible in field studies, including the elucidation of diagnostic biomarkers, correlates of protection, and mechanisms of vaccine-induced protection (8). Based on previous dose finding experiments, an inoculum deliberately providing less than 100% rate of infection (attack rate) in the control group was used to enable the calculation of vaccine efficacies (9). The attack rates in the present study were 77% in the control group, 35% in the ViTT group, and 37% in the ViPS group (2). Transcriptomics analysis has been shown to be highly effective when coupled with CHIM studies in observing host responses to vaccination and infection (10, 11). Here, we applied transcriptomics to investigate the divergent molecular signatures elicited by ViPS and the glycoconjugate ViTT in a randomized, phase IIb, controlled *Salmonella enterica* serovar Typhi CHIM (2).

Glycoconjugate vaccines are more immunogenic than plain polysaccharide vaccines and, importantly, are able to confer protection in infants and children (12, 13). Although the mechanism is still not fully defined, conjugation of T cell–independent polysaccharides to immunogenic carrier proteins stimulates the recruitment of CD4^+^ T cells, resulting in germinal center formation, which provides strong humoral and memory responses toward the polysaccharide antigen (12, 14). Here, we present a curated set of analyses describing changes in molecular signatures after vaccination and after challenge, comparing differences between vaccine groups and between participants who did not develop typhoid after exposure to the bacteria (non–typhoid-diagnosed [nTD]) and susceptible individuals who did develop typhoid fever (typhoid diagnosed [TD]). These analyses involve gene expression profiling methods, including differential gene expression analysis, gene set enrichment analysis (GSEA), time-course analysis, and weighted gene correlation network analysis (WGCNA), to describe overall transcriptomic changes across different time points, vaccine groups, and challenge outcomes; B cell receptor (BCR) repertoire analysis to quantify clonal expansion after vaccination; and a third complementarity-determining region 3 of the Ig heavy-chain–based (CDR H3–based) BCR clonotype clustering approach to identify biologically similar BCR clonotypes involved in Vi-polysaccharide binding at a range of time points after vaccination and *S*. Typhi oral challenge. Differential signatures between the Vi-containing typhoid vaccines were observed as early as 1 day after vaccination, as demonstrated by the higher gene perturbation in the ViTT group. The degree of gene perturbation increased further at day 7 after vaccination in the ViTT group, mainly driven by the upregulation of Ig genes. The expanded clonotypes at day 7 after vaccination (V7) showed that convergent humoral responses were elicited by the vaccines, while the dispersion of expanded clonotypes showed correlation with vaccine protection.

## Results

### Transcriptional response to typhoid vaccine and subsequent S. Typhi challenge.

**Figure 2:**
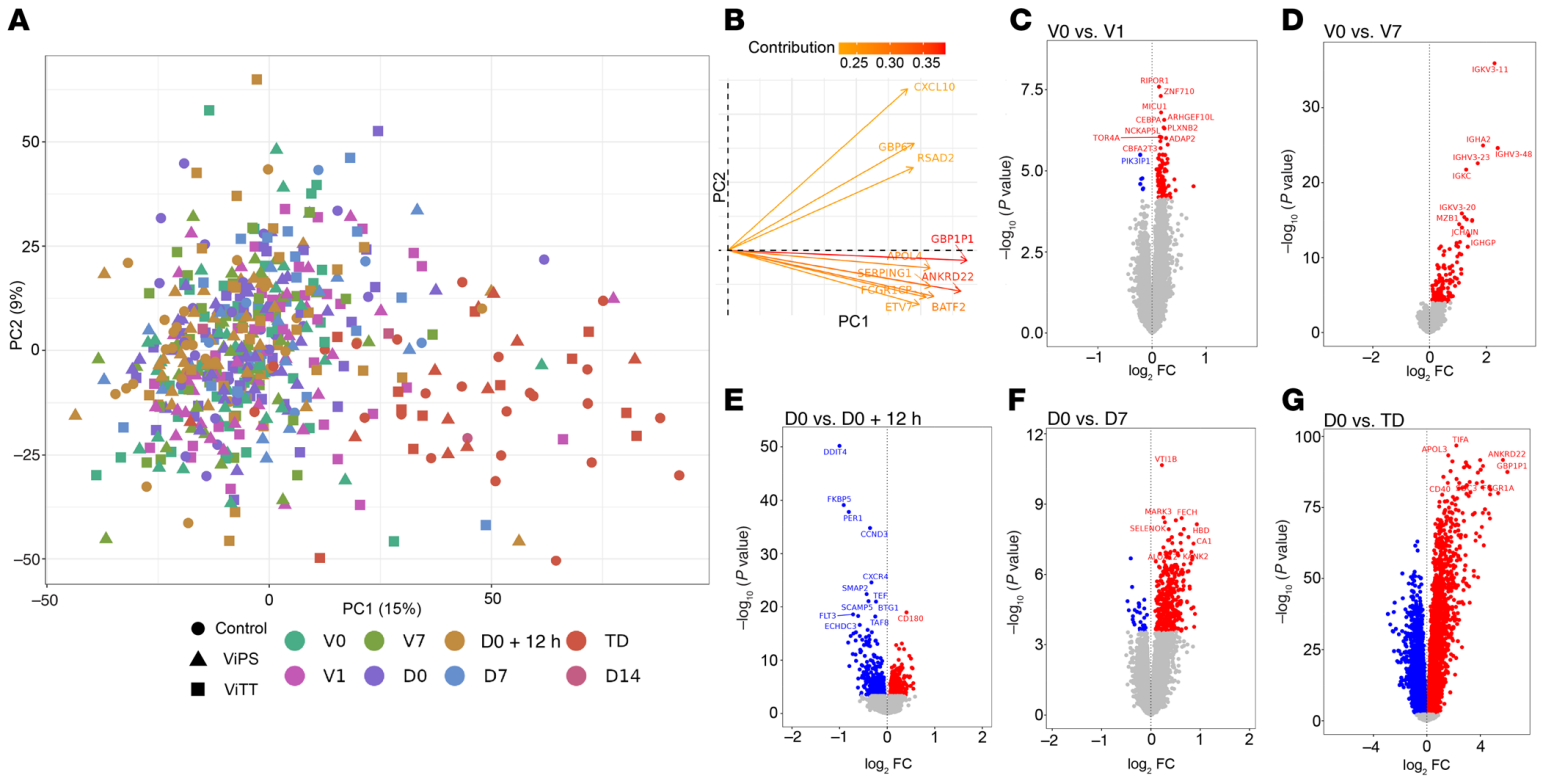
Global overview of blood gene expression data over the study time points. (**A**) PC analysis plot and contribution plot of RNA-Seq data (13,609 genes, *n* = 514) from all study time points, batch corrected for the sequencing pool. (**B**) Contribution plot of the genes contributing to PC1 and PC2. Genes with the greatest contribution are highlighted in red. (**C**) Volcano plot highlighting DEGs (FDR <0.01; red = upregulated, blue = downregulated) at day 1 after vaccination compared with pre-vaccination expression (99 DEGs, *n* = 72). (**D**) Volcano plot highlighting DEGs (FDR <0.01; red = upregulated, blue = downregulated) at post-vaccination day 7 compared with pre-vaccination expression (140 DEGs, *n* = 67). (**E**) Volcano plot highlighting DEGs (FDR <0.01; red = upregulated, blue = downregulated) at 12 hours after challenge compared with pre-challenge expression (678 DEGs, *n* = 101). (**F**) Volcano plot highlighting DEGs (FDR <0.01; red = upregulated, blue = downregulated) at day 7 after challenge (in the non-diagnosed group) compared with pre-challenge expression (172 DEGs, *n* = 50). (**G**) Volcano plot highlighting DEGs (FDR <0.01; red = upregulated, blue = downregulated) at typhoid diagnosis compared with pre-challenge expression (6,854 DEGs, *n* = 47). *P* values were obtained from the moderated *t* statistic, after adjustment for multiple testing (Benjamini and Hochberg’s method). The top 10 genes, ranked by FDR, are labeled.

Whole blood RNA-Seq was performed using samples collected at several time points of the study (Figure 1 and Table 1). Principal component (PC) analysis of the blood transcriptome from all study time points showed clustering — on the first PC — of samples taken at typhoid diagnosis (Figure 2A). *GBP1P1* and *ANKRD22* were identified as the genes with the greatest contribution to this clustering (Figure 2B). Differentially expressed genes (DEGs) were observed at all the study time points, with the peak in gene perturbation occurring at typhoid diagnosis (Figure 2, C–G). Differential gene expression comparison at the baseline showed no significant difference between nTD and TD participants, or between ViTT and ViPS recipients.

### Differences in early IFN signaling following conjugate typhoid vaccine compared with plain polysaccharide recipients.

**Figure 3:**
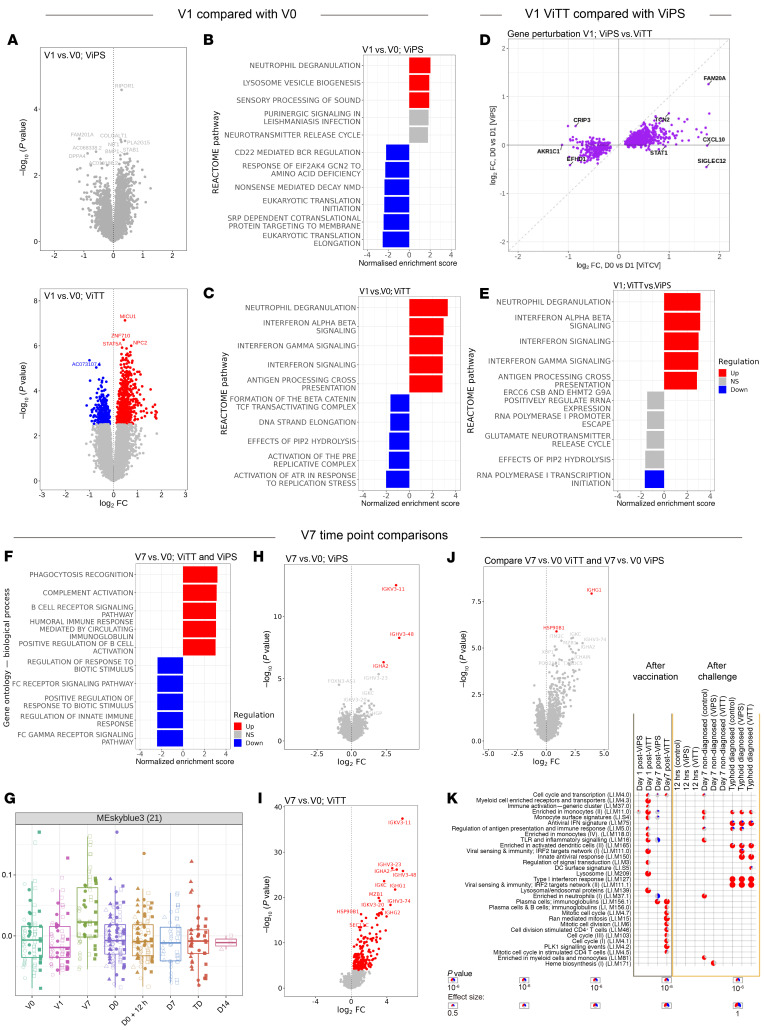
Blood gene expression profile on V1 and V7 for ViTT and ViPS recipients. (**A**) Volcano plot of changes in blood gene expression in ViPS or ViTT recipients at V1 compared with V0. (**B**) Top 5 upregulated and downregulated pathways from GSEA for ViPS recipients at V1 compared with V0. (**C**) Top 5 upregulated and downregulated pathways from GSEA for ViTT recipients at V1 compared with V0. (**D**) Agreement plot of fold change of DEGs (*P* < 0.05) for ViPS recipients at V1 (*y* axis) and ViTT recipients at V1 (*x* axis) compared with V0. (**E**) Top 5 upregulated and downregulated pathways from GSEA after ViTT vaccination compared with ViPS at V1. (**F**) Top 5 upregulated and downregulated pathways from GSEA at V7 for both Vi vaccine groups compared with V0. (**G**) A module derived from WGCNA that is associated with the V7 time point. (**H**) Volcano plot of changes in blood gene expression for ViPS recipients at V7, compared with V0. (**I**) Volcano plot of changes in blood gene expression in ViTT recipients at V7 compared with V0. (**J**) Volcano plot of differences in gene expression for ViTT recipients compared with ViPS recipients, both at V7. (**K**) Modular signatures induced during different study time points; enriched modules (FDR <1 × 10^–6^) are displayed. Segments of the pie charts represent the proportion of upregulated (red) and downregulated (blue) genes (absolute fold change >1.25).

We observed greater gene perturbation 1 day after vaccination in recipients of ViTT (154 DEGs) compared with those who received ViPS (no DEGs) (Figure 3A). GSEA found several differentially regulated pathways 1 day after vaccination, with the most significant term being “neutrophil degranulation” for both vaccine groups (Figure 3, B and C). Moreover, general agreement was seen in terms of the direction of gene regulation 1 day following either vaccine (Figure 3E). While at the gene level no statistically significant differential expression was discernible between the vaccine groups 1 day after vaccination, genes in the IFN signaling pathway, such as *STAT1* and *CXCL10*, were exclusively upregulated following ViTT (Figure 3D). Additionally, GSEA revealed several pathways that were differentially regulated between the vaccine groups 1 day after vaccination including the IFN signaling pathway (Figure 3E).

### Humoral immunity signature is present in blood transcriptome 7 days after typhoid vaccination.

The transcriptomic signature identified from whole blood 7 days after typhoid vaccination was characteristic of humoral immunity and BCR signaling (Figure 3F). Weighted gene correlation network analysis (WGCNA) described a gene module — composed of several Ig genes — that was prominent 7 days after vaccination (Figure 3G and Supplemental Figure 1, A and B). When ViPS and ViTT vaccine groups were analyzed separately, gene regulation 7 days after vaccination was qualitatively consistent, but quantitative differences were apparent (Figure 3, I and D, and Supplemental Figure 1C). Greater gene perturbation was observed 7 days after ViTT (186 DEGs) compared with ViPS (3 DEGs) (Figure 3, H and I). Two genes (*HSP90B1* and *IGHG1*) showed significantly greater expression 7 days after ViTT administration compared with the same time after ViPS administration (Figure 3J). Moreover, modular analysis described enrichment of genes associated with plasma cells for both vaccine groups at V7, and gene modules such as stimulated CD4^+^ T cells was exclusively enriched in ViTT recipients (Figure 3K), which is consistent with a T cell–dependent response. Increases in plasma cell signals were observed for ViTT recipients at V7 compared with ViPS recipients (Supplemental Figure 2).

Time-series analyses for post-vaccination time points identified 165 genes that were differentially regulated between nTD ViTT and ViPS recipients (Supplemental Figure 3). Several Ig heavy-chain variable region (*IGHV*) genes were present in the DEG list including the Ig genes *IGHV3-20*, *IGHV3-23*, *IGHV3-48*, and *IGHV3-74* (Supplemental Figures 4 and 5), aligning with the top DEGs at V7 in ViTT recipients compared with baseline (Figure 3J).

### BCR clonal expansion after vaccination is associated with protection against typhoid challenge.

**Figure 4:**
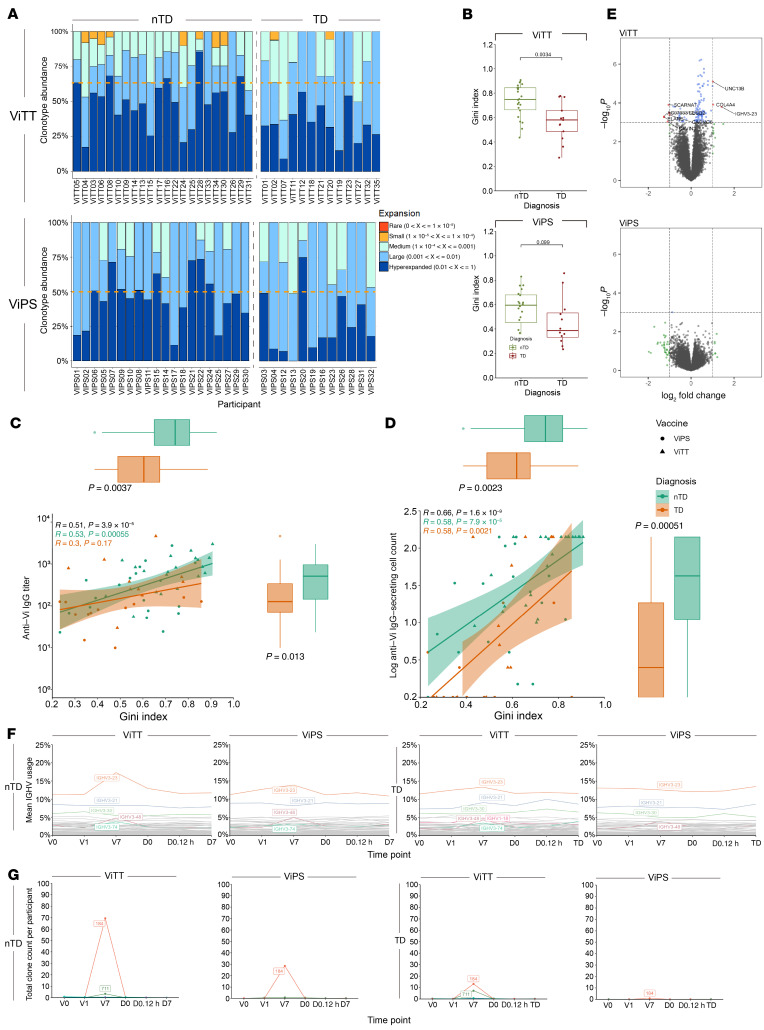
Clonal expansion in ViPS and ViTT participants at V7 with differential IGHV usage. (**A**) Clonal expansion at V7 measured by clonal space homeostasis. Clonotype abundance of 50% is indicated by an orange dotted line. (**B**) Gini index at V7. Significance was determined by Mann-Whitney *U* test. (**C** and **D**) The Gini index at V7 correlates with ELISA and ELISPOT data. *P* values for R values were calculated using Spearman’s rank correlation test. *P* values for the Gini index and total IgG titers were calculated using the Mann-Whitney *U* test. Box plots show the median and IQR of the Gini index, Vi-specific IgG titers, and log Vi–specific IgG-secreting cell count for nTD (green) and TD (orange) participants. (**C**) The Gini index at V7 correlates with Vi-specific IgG titers at D0. (**D**) The Gini index at V7 correlates with log Vi–specific IgG-secreting cells at V7. Participants who received ViPS are denoted by a circle, and participants who received ViTT are denoted by a triangle. nTD participants are labeled in green and TD participants in orange. (**E**) Volcano plot shows the differential gene expression profile of nTD versus TD participants in both the ViTT and ViPS groups at V7. (**F**) *IGHV* usage across the time points for nTD and TD participants who received ViTT or ViPS. Mean IGHV usage of each IGHV gene represents the mean of the percentage of total BCR clonotypes utilizing the IGHV gene for each participant. *IGHV* with greater than 5% usage or greater than 1% increase from V0 to V7 are highlighted in color. (**G**) Average total BCR count in participants who expressed the BCR cluster.

Using the upregulation of *IGHV* genes as a guide, further examination of *IGHV* gene usage in response to vaccination was performed for the BCR repertoire of Vi vaccine recipients. The magnitude of BCR clonal expansion was initially quantified with the proportion of the total repertoire occupied by each clonotype, hence clonal space homeostasis, using Immunarch (15) (Figure 4A). The result showed that at V7, half (11 of 22) of ViTT nTD participants had a peripheral B cell repertoire composed of 50% or more hyperexpanded clonotypes, compared with 15% (2 of 13) for TD participants. Interestingly, 40% (8 of 20) of nTD ViPS recipients also showed B cell repertoires of 50% or more hyperexpanded clonotypes at V7, whereas only 1 of 12 TD ViPS recipients showed this level of hyperexpanded clonotypes.

To quantify clonal expansion, the Gini index was calculated for each participant and compared between vaccine groups and challenge outcomes. A significant difference was observed when contrasting the Gini index between nTD and TD participants who received ViTT; nTD participants underwent a significantly more robust clonal expansion compared with TD participants (Figure 4B). However, no significant difference in the Gini index was observed between ViPS recipients with different challenge outcomes (Figure 4B). In addition, as the hyperexpansion of clonotypes may correlate to a higher antibody titer at later time points, we correlated the Gini index with Vi-specific IgG titers at the day of challenge (D0) (Figure 4C). Significant correlations were observed between the Gini index at V7 and Vi-specific IgG-secreting cell counts at V7 (Figure 4D).

### IGHV3-23 usage is associated with protection following typhoid challenge.

Differential gene expression analysis revealed upregulation of *IGHV3-23* in ViTT nTD participants at V7 compared with V0. However, no similar upregulation of *IGHV* gene expression was observed for ViPS recipients (Figure 4E). To investigate BCR gene usage at the repertoire level between ViTT participants with different challenge outcomes, we retrieved and filtered the CDR H3 sequences using MIXCR and VDJ tools. In total, 90,574 unique BCR sequences were identified for ViTT nTD participants across all time points, and 43,057 were identified for ViTT TD participants. Among all BCR sequences retrieved from ViTT nTD participants at V7, 17.30% used *IGHV3-23*, followed by *IGHV3-21* (8.24%) and *IGHV3-30* (5.48%) (Figure 4F). In contrast, ViTT TD participants at V7 showed a more mixed profile comprising *IGHV3-23* (13.35%), *IGHV3-21* (9.13%), and *IGHV3-30* (5.99%) (Figure 4F). Among the ViPS nTD participants, *IGHV3-23* had the highest usage at V7 (13.87%), followed by *IGHV3-21* (8.16%) and *IGHV3-30* (4.93%). ViPS TD participants showed higher *IGHV3-23* usage at V7 (12.59%), followed by *IGHV3-21* (7.68%) and *IGHV3-30* (5.02%).

### CDR H3 clustering identifies amino acid sequence clusters that potentially contribute to Vi-polysaccharide binding.

*IGHV3-23*, like other *IGHV* genes, encodes a sequence that partially spans the complementarity-determining region H3 (CDR H3) loop. BCR clonotypes with similar CDR H3 sequences and V, D, and J gene usages may confer similar binding capabilities (16, 17). Thus, with the aim of identifying the definitive biological mechanism underlying the protection derived from *IGHV3-23* upregulation, BCR sequences using IGHV3-23 by amino acid similarities were clustered using a Hamming distance-based clustering approach. In total, 24,974 unique clusters were identified across the combined data sets for ViTT, ViPS, and controls. Of these clusters, 12,928 were identified in ViTT recipients, 8,474 were identified in ViPS recipients, and 3,987 unique clusters were identified in control vaccine recipients. After assigning all unique clones with cluster numbers, filters were applied to (a) display clusters present in more than 25% of ViTT participants and (b) display clusters with no single participant expressing 50% or more of the total count. These filters were applied to identify convergent clonotypes between participants that may contribute to protection (Figure 4G). From this analysis, cluster 184 was shown to be the most commonly upregulated cluster at V7 in both ViTT and ViPS nTD participants. In particular, cluster 184 was upregulated in nTD participants compared with TD participants. The total clone count normalized by the number of participants expressing clonotypes within this cluster for ViTT nTD, ViTT TD, ViPS nTD, and ViPS TD participants was 69.50, 13.15, 28.50, and 0.83, respectively (Figure 4G).

**Figure 5:**
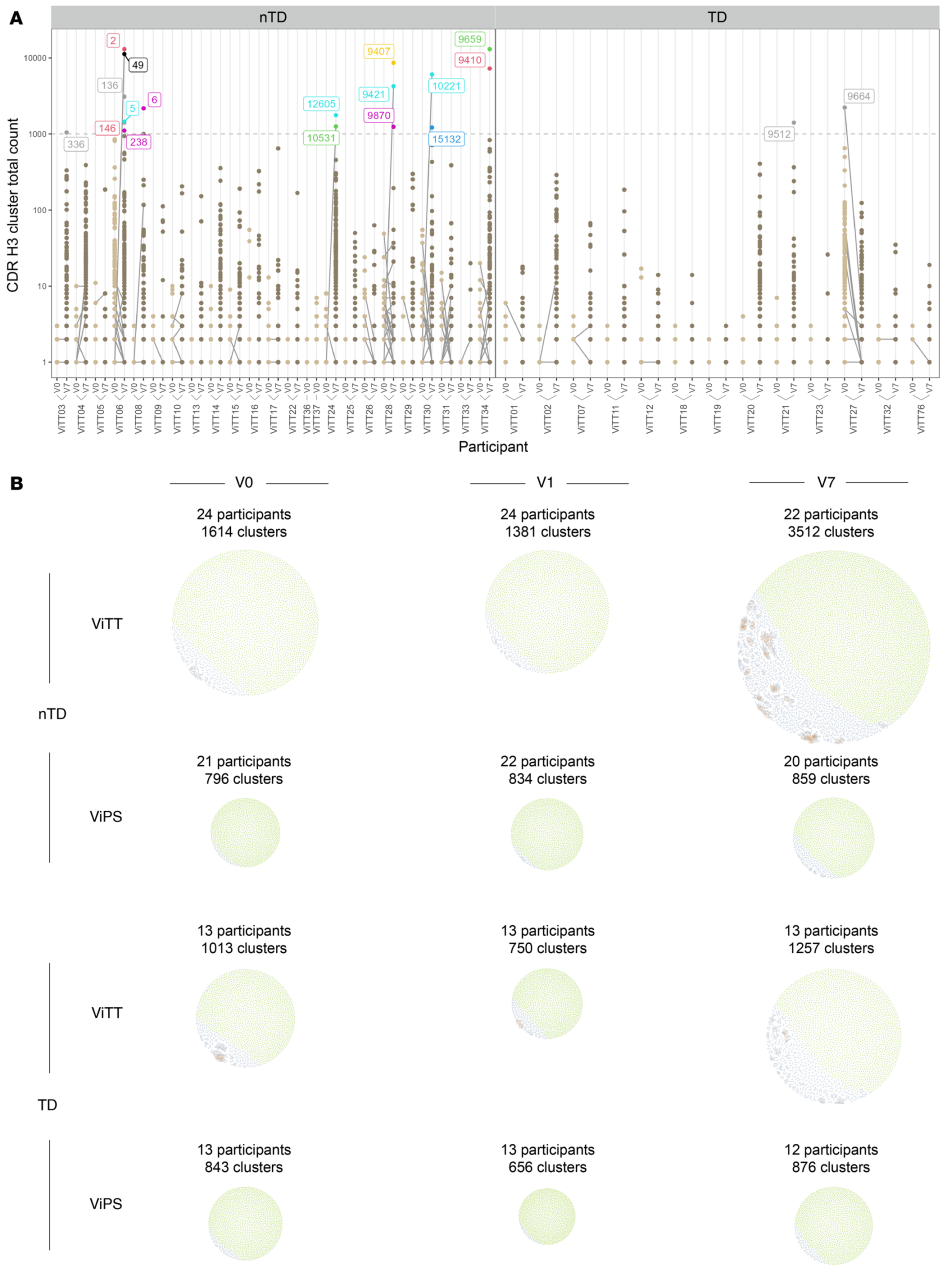
Visualization of BCR clusters. (**A**) BCR clusters in participants receiving ViTT. Clusters with a total count of 1,000 or higher are shown in individual colors. Data from V0 are shown in light brown, and data from V7 are shown in darker brown. The same clusters between V0 and V7 are connected by gray lines. (**B**) BCR clusters generated from participants receiving ViPS or ViTT at V0, V1, and V7 time points presented as network plots. Groups were separated by challenge outcome. The size of the network corresponds to the number of clusters within it, with each cluster represented by a dot. Clusters that comprise 1 unique BCR clonotype are shown in green, and clusters that comprise more than 1 unique BCR clonotype are shown in blue. Clonotypes within clusters that are connected to more than 6 other sequences in the cluster are shown in amber to help locate the center of each cluster.

The nTD participants are characterized by the upregulation of several clusters that were unshared between participants. These clusters may represent individual responses to the vaccines (Figure 5A). In general, the number of clusters identified was higher in the ViTT group than in the ViPS group, whereas the same was observed when comparing the nTD group with the TD group (Figure 5B).

**Figure 6:**
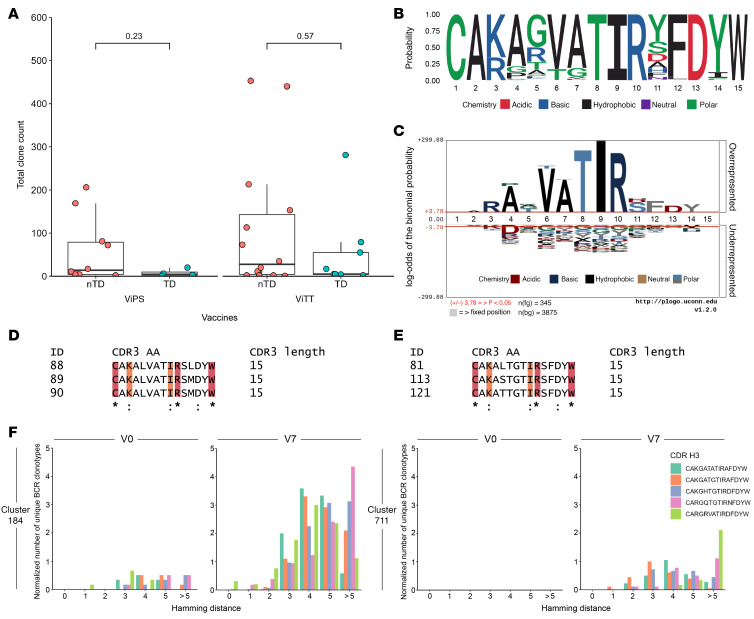
Amino acid sequence conservation of BCR cluster 184. (**A**) Expression of cluster 184 in ViTT and ViPS recipients at V7. (**B**) Logo plot highlighting probability and amino acid residue properties. (**C**) pLogo plot demonstrating that most amino acid residues are significantly outstanding from the background. Significance was determined by Mann–Whitney U test; the red lines indicate a significance level cutoff of *P* < 0.05. (**D** and **E**) Amino acid sequence conservation calculated by Clustal Omega (19) between (**D**) cluster 184 CDR H3 sequences alone and (**E**) cluster 184 CDR H3 sequences and known Vi-binding BCR CDR H3 sequences. Three example sequences for each are included for simplicity. An asterisk denotes positions that have a single, fully conserved residue; a colon denotes amino acids with strong similarity, which scores higher than 0.5 in the Gonnet PAM 250 matrix. Conservation is highlighted in crimson (*) and orange (:). (**F**) Unique BCR clonotypes in cluster 184 and 711 and the relative proximity to known Vi-binding BCR CDR H3 sequences at V0 and V7 for ViPS and ViTT participants. The number of unique BCR clonotypes was normalized by the number of participants for each time point.

Cluster 184, composed of 345 unique B cell clonotypes, was shared between 16 of 24 nTD and 11 of 13 TD participants receiving ViTT. The cluster was also shared between 12 of 22 nTD and 5 of 13 TD participants receiving ViPS. Comparison of cluster expression at V7 for both vaccine groups showed that the highest expression was in the nTD group (Figure 6A). Alignment of all cluster 184 CDR H3 sequences showed a high level of CDR H3 conservation (Figure 6B). When compared with the background, as defined by all other CDR H3 sequences from unique clones using IGHV3-23, the conservation was statistically significant (Figure 6C). Of the 15–amino acid CDR H3 sequence, the conservation of the TIR motif at positions 8–10 was the most significant compared with the background. This motif was searched against the CDR H3 sequences of known Vi-binding antibodies (18). Six Vi-binding antibodies were identified as comprising 15–amino acid–long CDR H3 regions while also comprising IGHV3-23, with 2 comprising identical CDR H3 sequences. To validate the potential binding capabilities of this cluster, alignment with CDR H3 sequences was performed (a) between sequences within the cluster and (b) between sequences within the cluster and the 6 additional CDR H3 sequences from Vi-binding antibodies (18). Both alignments returned the same amino acid conservation annotation from Clustal Omega (19), implicating potential Vi-polysaccharide binding (Figure 6, D and E). There was no significance observed when comparing expression on an individual level (Figure 6A). Comparison was also made between the 6 Vi-binding clonotypes with clusters 184 and 711 to determine the Hamming distance between each clonotype within the cluster with the CDR H3 sequences of known Vi-binding antibodies. We observed that both clusters were highly similar to the CDR H3 sequence of known Vi-binding antibodies at V7 (Figure 6F).

### BCR clonal expansion in ViTT recipients is not primarily directed toward the carrier protein.

BCR clonal expansion was observed in ViTT recipients at V7 (Figure 4, A and B). As both the carbohydrate and peptide moieties of the vaccine are viable ligands to BCRs, it was of interest to assess which moiety the expansion of BCR clonotypes was primarily directed toward to understand the makeup of ViTT-induced humoral immunity. A total of 667 known TT-binding B cell clonotypes were retrieved from the literature, of which the *IGHV* usage in 317 clonotypes was known. Of these clonotypes, 16 of 317 comprised *IGHV3-23* (20–29). In contrast, our knowledge of Vi-polysaccharide–binding clonotypes comprised 52 clonotypes previously described, of which 20 of 52 used IGHV3-23 (18). To assess the nature of clonal expansion after ViTT vaccination, the total number of unique B cell clonotypes incorporating *IGHV3-23* was divided by the total number of likely TT-binding clonotypes identified in ViTT participants at V7. Likely TT-binding clonotypes were defined by the clonotypes that carry CDR H3 sequences within 1 amino acid difference of known TT-binding clonotypes, while keeping the same CDR H3 length. The results showed disproportional upregulation of clonotypes comprising *IGHV3-23* compared with likely TT-binding clonotypes (Supplemental Figure 6). To validate the presence of likely TT-binding CDR H3 sequence as a ViTT-specific response, the total expression of BCR clonotypes containing these likely TT-binding CDR H3 sequences was compared between ViPS and ViTT participants, with significantly higher expression observed in ViTT recipients 7 days after vaccination (Supplemental Figure 7).

### Typhoid challenge is associated with early changes in gene expression that peak at the time of typhoid diagnosis.

**Figure 7:**
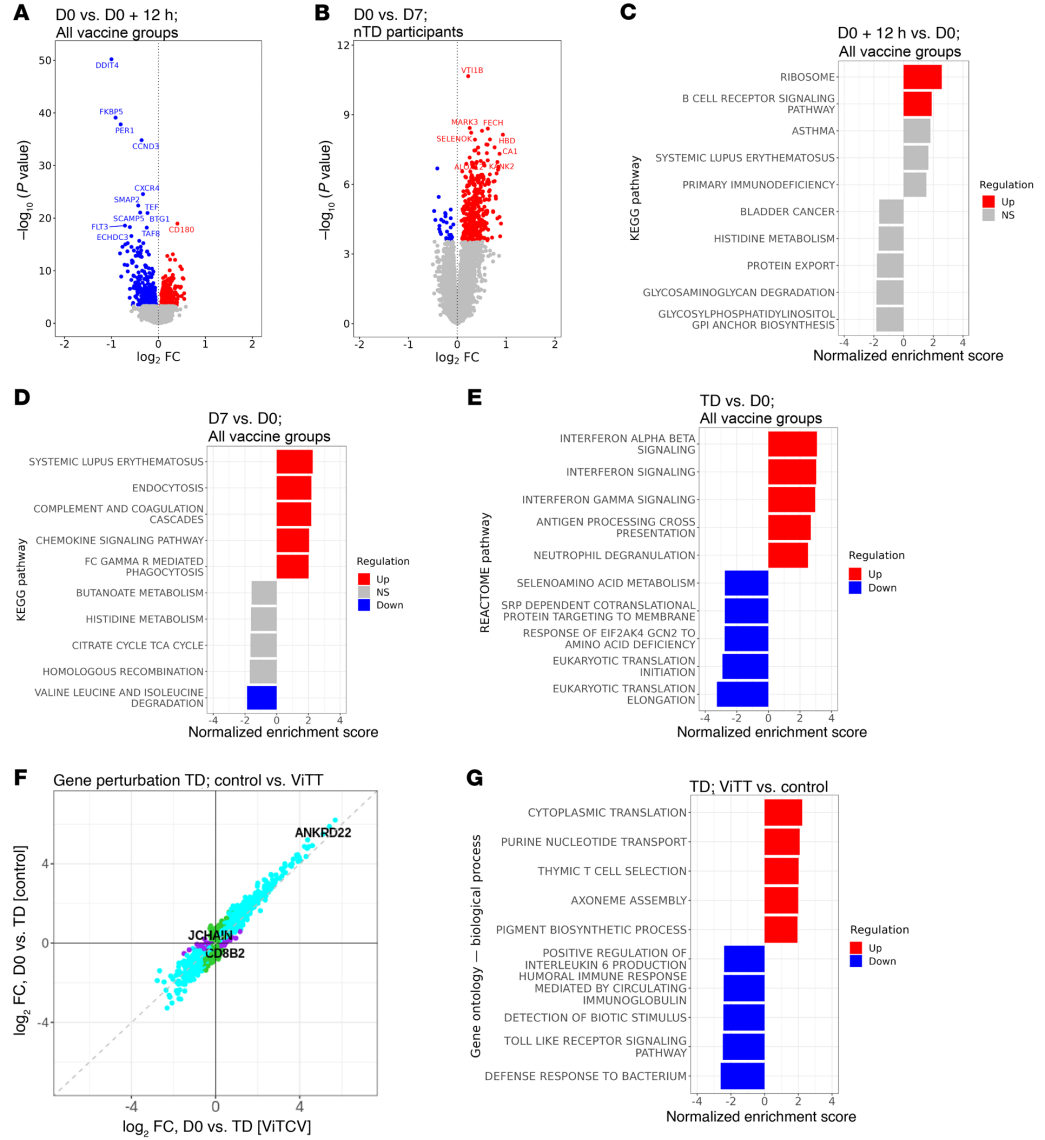
Post-challenge blood gene expression profile for ViTT and ViPS recipients. (**A**) Volcano plot of differences in gene expression on D0+12h compared with D0 for all participants. (**B**) Volcano plot of differences in gene expression on D7 compared with D0 for participants who did not develop typhoid fever. (**C**) The top 5 upregulated and downregulated pathways from GSEA on D0+12h. (**D**) The top 5 upregulated and downregulated pathways from GSEA on D7 for those who did not develop typhoid fever. (**E**) Top 5 upregulated and downregulated pathways from GSEA at the day of typhoid diagnosis compared with day of challenge. (**F**) Agreement plot of changes in gene expression (DEGs only) at typhoid diagnosis in those who received ViTT compared with control vaccine recipients. (**G**) Top 5 upregulated and downregulated pathways from GSEA at the day of typhoid diagnosis for ViTT versus control vaccine recipients.

To understand the interplay between vaccines and *S*. Typhi challenge, a series of differential gene expression analyses was performed. As early as 12 hours after challenge, we observed changes in blood gene expression including increases in CD180, a cell-surface molecule involved in B cell recognition of LPS. GSEA also indicated upregulation of the BCR signaling pathway at this early time point (Figure 7, A and C). Measurable differences (DEGs = 390) in the blood transcriptome of nTD participants were observed at D7 compared with the baseline (Figure 7B). These changes were enriched for genes associated with pathways such as those for complement cascades and Fc γ receptor–mediated phagocytosis (Figure 7D). In this study, the peak of gene perturbation was seen at typhoid diagnosis (6,854 DEGs; Figure 7F and Supplemental Figure 8). At typhoid diagnosis, IFN signaling, antigen-processing, and neutrophil degranulation pathways were enriched (Figure 7E). The gene perturbation was largely similar at typhoid diagnosis regardless of the vaccine received (Figure 7F and Supplemental Figure 9D). However, differences in gene regulation at the pathway level were evident in individuals who previously received a typhoid vaccine, including differences in the regulation of T cell pathways in ViTT recipients (Figure 7, F and G).

### Attenuated gene perturbation after infection in ViTT recipients compared with other groups: time series analysis.

**Figure 8:**
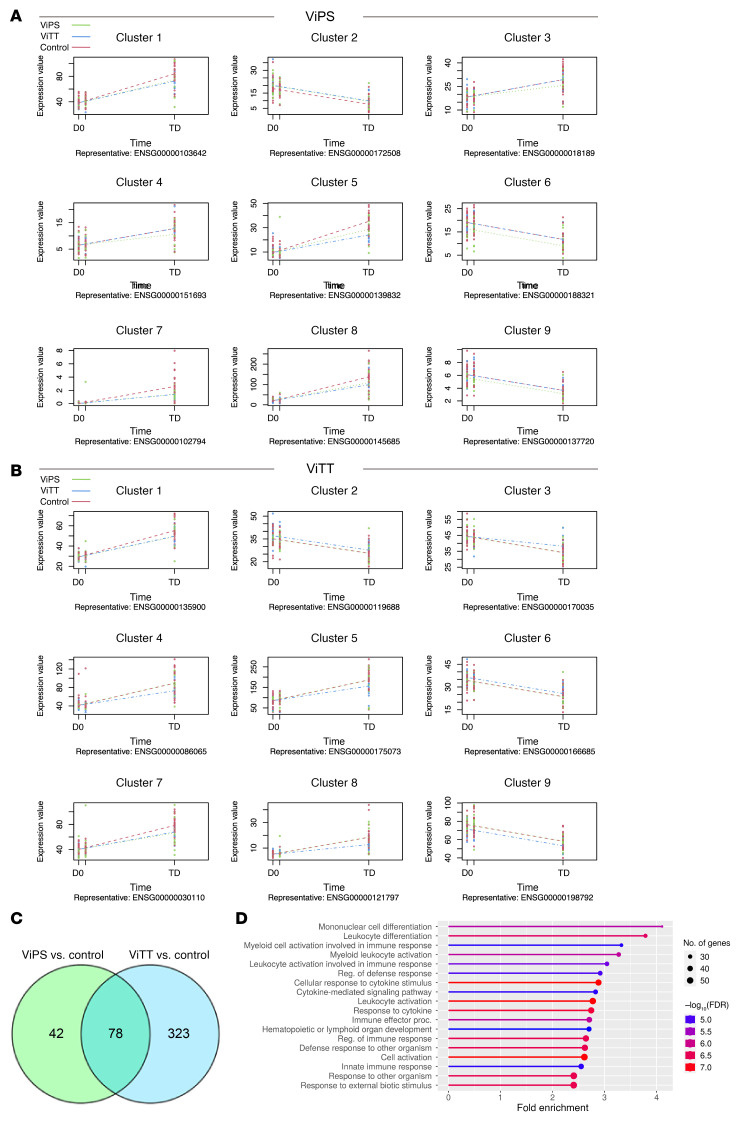
Significantly different expression profiles across time points following challenge. (**A**) For participants vaccinated with ViPS, a representative example gene from each cluster is plotted. Regression fit curves are shown for each group. An expression profile of these genes for ViTT recipients is also shown as a reference. (**B**) For participants vaccinated with ViTT. a representative example gene from each cluster is plotted. Regression fit curves are shown for each group. An expression profile of these genes for ViPS recipients is also shown as a reference. (**C**) Overlap between the significantly expressed genes in ViPS and ViTT vaccination groups compared with control. (**D**) Significantly enriched GO terms (biological process) among 323 genes with significantly different expression profiles for the ViTT-vaccinated group but not the ViPS-vaccinated group.

Next, a time series analysis was performed on all TD participants for the post-challenge time points. Both ViPS- and ViTT-vaccinated participants had a sizable set of genes (120 and 401, respectively) that showed different trends across the time points following challenge compared with the control group (Figure 8, A and B). The genes with similar profiles were grouped together in a cluster, most of which showed decreased gene expression perturbation in ViTT and ViPS recipients following challenge and at the TD time point compared with the control group.

Despite the fact that both Vi vaccine groups revealed 9 clusters of significant genes of genes with significant expression profile differences, only 2 clusters (clusters 3 and 4) showed more distinct trends in ViPS-vaccinated participants compared with the ViTT-vaccinated and control groups. Three other clusters showed the same direction of change for both Vi vaccines compared with the control group (clusters 1, 7, and 8) (Figure 8A). The remaining clusters were biased in overall expression across the participants in the ViPS group and therefore could not be used to interpret the response to the challenge (clusters 2, 6, and 9). None of the clusters revealed significant enrichment of gene ontology (GO) terms. On the other hand, ViTT recipients had a more distinct profile than did the other 2 groups (Figure 8B). Clusters 4, 5, and 8 showed a lower degree of perturbation in ViTT participants at TD, with only cluster 3 showing increased perturbation. GO terms analysis of the genes in those clusters revealed enrichment of multiple biological processes.

Both Vi vaccine groups responded differently to typhoid infection compared with the control group: ViPS and ViTT recipients share 78 genes that were significantly differentially expressed, whereas 42 and 323 genes were uniquely differentially expressed in the ViPS group and ViTT group, respectively (Figure 8C). GO terms enrichment analysis of those genes that were unique to the ViTT group revealed enrichment of genes in multiple pathways involved in immune responses, such as the cytokine response, leukocyte activation, and the innate immune response (Figure 8D).

Further analysis performed on the clinical data around typhoid diagnosis showed that ViTT recipients had lower median C-reactive protein (CRP) levels after diagnosis of typhoid fever (Supplemental Figure 10A). Fever over 38°C was present in 23% of the ViTT-vaccinated participants compared with 36% of the ViPS participants and 46% of the control group (Supplemental Figure 10B). However, none of the observed clinical differences between the groups was statistically significant (Mann-Whitney *U* test, *P* < 0.05).

## Discussion

Despite previous attempts to identify correlates of protection following Vi-derived vaccines in both CHIM and field trials, the correlates of protection against typhoid fever remain unclear (2, 3, 30). Here, we used contemporary methods to present a high-resolution molecular description of responses to vaccination and the subsequent response to infection. The endpoint of this study showed that the percentage of participants protected by ViTT (65%) and ViPS (63%) was higher than the background resistance to infection in the control vaccine recipients (23%) (2). To elucidate the protective mechanisms of these vaccines, features of the transcriptomic response contributing to protection were identified. In spite of the wide array of results generated in the present study using whole blood bulk RNA-Seq data, this sequencing approach lacks the information regarding which cell types express the transcripts. The cellular heterogeneity is masked in bulk RNA-Seq, hence preventing the deconvolution of the cell types. It has been previously documented that higher levels of preexisting *S*. Typhi–responsive CD8^+^ memory T cells are associated with an elevated risk of typhoid acquisition (31). Hence, further analyses that utilize single-cell sequencing approaches may be of interest to provide insights into the molecular signatures associated with particular cell types.

From the PC analysis conducted for all time points, we observed that expression of *GBP1P1* and *ANKRD22* afforded the greatest amount of clustering. *GBP1P1* represents a pseudogene of guanylate-binding protein 1 (GBP1), which is responsible for autophagosome formation during intracellular pathogen infection (32). This is in accordance with the intracellular properties of *S*. Typhi (33), implying that PC analysis signals may be centered around gene perturbations as a result of the *S*. Typhi challenge. Similarly, upregulation of *ANKRD22* was shown to be associated WT I and type III IFN production (34), which aligns with the initial observations of IFN signaling after vaccination and after challenge. When individual time points were assessed, we observed early responses 1 day after vaccination that were associated with neutrophil degranulation in both the ViPS and ViTT groups. Enrichment of genes connected to neutrophils and neutrophil function have previously been described in blood following the administration of other vaccines (35, 36). This early gene signature probably reflects the rapid recruitment of neutrophils to the site of vaccination (37). Interestingly, while broad agreement was seen between the vaccine groups in terms of gene expression 1 day after vaccination, GSEA revealed several pathway genes that were differentially regulated between these groups including IFN signaling pathway genes. For example, genes in the IFN signaling pathway such as *STAT1* and *CXCL10* were exclusively upregulated after ViTT. Following vaccination, antigen can reach draining lymph nodes (dLNs) within hours, either through diffusion of soluble antigen or via transportation by neutrophils or monocytes (37). Therefore, the IFN signaling pathway stimulation seen in the blood could be propagated by vaccine site inflammation or by early immune responses in the dLNs. In mice, IFN-related genes are modulated in local muscle as early as 6 hours after vaccination (38). Acute inflammation and innate immune pathways are also promptly initiated in the dLNs, with recruitment of neutrophils and monocytes within 24 hours of vaccination and the upregulation of IFN-inducible genes (38, 39). These early transcriptomic differences between the vaccine groups may be due to rapid recognition of the conjugate carrier protein — all individuals will have existing immunity to TT — by memory T cells. This can take place in the tissue, as effector memory cells can reside within tissue or migrate to the site of inflammation (40). Alternatively, this recognition may take place in the dLNs, where central memory T cells localize (41). Apart from the upregulation of genes observed 1 day after vaccination, another molecular signature observed when comparing all participants at V1 with the baseline was the significant downregulation of phosphatidylinositide 3 kinase–interacting protein 1 (*PIK3IP1*). *PIK3IP1* is highly expressed in naive T cells, whereas its downregulation has been associated with T cell activation, which may indicate that the glycoconjugate vaccine was inducing T cell activation beginning on day 1 after vaccination (42).

Robust upregulation of B cell–related transcriptomic profiles was observed 7 days after vaccination in both ViPS and ViTT recipients. The viability of using the day-7 post-vaccination time point to investigate humoral responses can be based on previous observations that antibody-secreting cell levels rise 7 days after vaccination, while total IgG levels peak 2–28 days after Vi-derived vaccine administration (6, 43). Similar observations were documented for both plain polysaccharide-based vaccines and glycoconjugate vaccines against meningococcal infections, in which the total serum IgG titers and PBMC counts rise rapidly between 7 and 30 days after vaccination (44, 45). Despite the fact that upregulation was observed following both types of vaccines, the magnitude of this upregulation was greater in the ViTT group, as demonstrated by a higher fold change in the expression of several Ig light- and heavy-chain variable region genes and upregulation in gene modules related to plasma cells and Ig production. While this observation is in line with previous reports (44), it is also in agreement with the T cell–dependent properties of glycoconjugate vaccines, which induce affinity maturation and clonal expansion. The T cell–dependent mechanism is also reflected by the upregulation of CD4^+^ T cell and cell division–related gene modules in the ViTT group but not in the ViPS group. In contrast, ViPS is T cell independent and does not induce affinity maturation (44, 46). Higher levels of Fc receptor signaling pathway activation in the ViTT group may also imply a more robust level of Fc-mediated functions, which has previously been correlated with vaccine protection against pathogens including HIV (47). Another notable difference between ViPS and ViTT at post-vaccination day 7 was the upregulation of *IGHG1* in the ViTT group. *IGHG1* upregulation has been widely observed as a vaccine response in humans following administration of protein-based vaccines (48–50), reflecting a humoral response dominated by the IgG1 subclass. The upregulation of *IGHG1* in ViTT recipients contrasts with the property of plain polysaccharide-based vaccines to induce humoral responses dominated by the IgG2 subclass (51, 52). It has been well documented that IgG1 can mediate more effective bactericidal and opsonic activities than IgG2 (53–55). Previous studies showed that serum IgG1 is important for the protection against typhoid and plays an important role in ViTT-induced protection (6, 56). In addition, our study showing differential expression profiles of gene clusters between nTD participants in ViTT and ViPS groups also suggests that the genes associated with protection differed between the vaccine groups, as demonstrated by the upregulation of gene clusters following vaccination in ViTT, but not ViPS, recipients.

Administration of glycoconjugate vaccines can induce the expansion of antigen-specific B cells mediated by helper T cells (57, 58). Clonal expansion of B cells has been widely observed after administering several types of T cell–inducing vaccines, including inactivated influenza vaccine, live attenuated yellow fever vaccine, and recombinant hepatitis B vaccine (59–62). These vaccine types have been known to recruit CD4^+^ T cells for germinal center formation and subsequent affinity maturation to produce large quantities of antibodies with high antigen-specificities (63, 64). We observed that in ViTT recipients, the disproportional expansion of BCR clonotypes resulted in the top few most expressed clonotypes comprising the majority of the BCR repertoire. This observation was reminiscent of the correlation seen between the level of B cell clonal expansion at day 7 after vaccination with the likelihood of seroconversion from an inactivated influenza vaccine (65). To assess this disproportional expansion of BCR clonotypes, the Gini index was used as a means of quantification. Significant differences were observed between the BCR repertoire of ViTT nTD participants and TD participants, correlating with vaccine-induced protection. A higher Gini index may suggest expansion of potentially high-affinity and -avidity BCR clonotypes as a result of affinity maturation. The correlation seen between the Gini index and anti-Vi IgG titers was consistent with previous descriptions that serum anti–Vi IgA and anti–Vi IgG1 levels and the antibody avidities were partially associated with protection against *S*. Typhi infection (6, 56).

We observed that specific Ig-related genes were upregulated after vaccination. This is in line with documentations of similar upregulations after vaccination with vaccines against several Gram-negative bacteria (58, 66). Notably, *IGHV3-23* was the most commonly used *IGHV* gene in nTD participants. CDR H3 clustering of clonotypes using *IGHV3-23* showed that, despite the expansion of both private and public repertoires in ViTT recipients, private repertoires tended to be more expanded than public repertoires. As a result of affinity maturation, there is good reason to expect that the expanded clonotypes are Vi binding. Despite being more prominent in ViTT recipients, cluster 184 emerged as the most commonly upregulated cluster in both vaccine groups 7 days after vaccination. This observation aligns with the comparable efficacies demonstrated by the 2 vaccines in this CHIM setting, while also being in line with the similar clinical and microbiological outcomes observed among the TD participants. However, it is also noteworthy that glycoconjugate and plain polysaccharide vaccines containing the same sugar component can induce the formation of distinctly different antigen-specific B cell repertoires (58). This may also suggest that investigating the private B cell repertoires of the vaccine recipients may aid in the identification of novel protective antibodies against typhoid fever.

To explore the properties of the clonotypes within cluster 184, CDR H3 sequences were compared with avid clonotypes identified previously (18). Exact CDR H3 matches alongside many highly similar clonotypes were observed. Previous binding analyses also showed that the matching clonotypes bind both *O-*acetylated and de-*O-*acetylated Vi polysaccharide, enabling them to mediate antibody-dependent Fc effector functions (18). It will be of future interest to model the binding interactions between these BCR clonotypes and Vi-polysaccharide. A more efficacious vaccine design can be proposed by identifying the binding partners of the paratopes present on these Vi-specific BCR clonotypes. Several computational tools are able to perform modeling of antibody-glycan interaction, including GlycoTorch Vina, Vina-Carb, and GlycanDock (67–69). However, while the output from these tools generally resembles partial or near-complete resemblance of the actual interaction between the antibody and the glycan, the results are varied between different antibody-glycan pairs (68). From these modeling analyses, specific moieties or certain lengths of repeat units of Vi-polysaccharide may be found to demonstrate preferential binding with the likely Vi-binding clonotypes compared with the native Vi-polysaccharide extracted from bacterial culture, and this may identify potentially more potent vaccine antigens to incorporate into vaccine design. Subsequently, the desired glycans can be chemically synthesized to produce highly reproducible products that can act as a standalone vaccine candidate or further derivatized into glycoconjugates (70). Considering that the chemical synthesis of Vi-polysaccharide has been demonstrated in the past (71, 72), improved vaccine design derived from the present readouts is likely feasible.

*IGHV3-23* is involved in producing antibodies that bind Vi-polysaccharide (18). The disproportional humoral response observed between BCR clonotypes using *IGHV3-23* and likely TT-binding clonotypes in nTD participants is reminiscent of the humoral response elicited by a pneumococcal glycoconjugate vaccine, with disproportional IgG3 antibody titers against the carrier protein and the polysaccharide moieties (73). Although the mechanism of the disproportional response is unknown, it may be related to the conformation in which antigen is presented on glycoconjugate vaccines. Glycoconjugate vaccines usually adopt either a “sun-type” conformation or a “lattice-type” conformation (14). Both conformations allow the carbohydrate moiety of the glycoconjugate to be exposed to BCR binding in the clonal selection process. The steric bulk created by the carbohydrate moieties would shield the carrier protein from BCR binding, making the affinity maturation and clonal expansion toward the carrier protein less likely (74, 75).

GSEA readouts suggest that B cell signaling and ribosome-related gene set upregulation are observable as early as 12 hours after challenge in all vaccine groups. This may in turn reflect early signals of the B cell memory response induced by the glycoconjugate vaccine, in which memory B cell differentiation has occurred (57). The median times for the typhoid diagnosis in each group were as follows: 6.0 days after challenge (IQR, 5.1–7.8) in the control group, 6.5 days (IQR, 6.1–8.6) in the ViTT group, and 7.2 days (IQR, 5.9–10.2) in the ViPS group. There were no statistically significant differences between the groups (Mann-Whitney *U* test, *P* < 0.05). Hence, analyses were performed to compare the molecular responses 7 days after challenge as the closest available time point in the nTD participants and TD participants. At day 7 after challenge, hence the D7 time point, gene sets related to endocytosis and Fc-γ receptor–mediated phagocytosis were highly upregulated in the nTD participants, which directly implicated the occurrence of antibody-dependent neutrophil phagocytosis (ADNP) within these participants. ADNP has been previously associated with protection against enteric fever (76). In contrast, TD participants at day 7 after challenge showed typical signals of typhoid pathogenesis represented by the upregulation of IFN signaling pathways (77).

The expression of several noteworthy genes was observed in the protected participants 7 days after challenge: *VTI1B* encodes a soluble NSF attachment protein receptor (SNARE) protein and was observed to be strongly upregulated in nTD participants 7 days after challenge. *VTI1B* can associate with another SNARE protein, syntaxin 6, to form a SNARE complex that mediates fusion events in secretory pathways, seen extensively in the trafficking of TNF-ɑ from activated macrophages (78). In addition, the upregulation of *FECH*, which encodes ferrochelatase, the final enzyme of the pathway involved in heme biosynthesis, was observed. The upregulation of *FECH* is often associated with hypoxic cells (79). While hypoxia can result from a range of factors, it is also a hallmark of inflammation (80). Taken together, these gene upregulations may indirectly implicate inflammation even in participants who were not diagnosed with typhoid after challenge. Enrichment of GO terms related to mononuclear cell differentiation and leukocyte enrichment in ViTT, but not in ViPS, recipients also agrees with the T cell–dependent property of ViTT.

Time series analysis of protected participants showed ViTT-specific gene profiles not evident in ViPS recipients. For example, the increased expression of the genes from unfolded protein response pathways was enriched uniquely in the ViTT group after vaccination. This pathway has been previously shown to be associated with a higher T cell response to influenza vaccine and may enhance antigen presentation to T cells (81, 82). In addition, in the ViTT recipients who developed typhoid following challenge, perturbation of the immune system appeared to be attenuated compared with the control group. This finding is in line with previous studies showing that even when vaccination does not result in protection from disease (6), multiple vaccines have been shown to reduce the bacteremia and symptoms (83). The responses to typhoid infection in ViTT recipients were characterized previously by reduced inflammation based on the reduction in symptom severity scores (6). Our additional analysis of the clinical data revealed lower CRP levels and a lower proportion of participants presenting with fever within the ViTT group at the time of diagnosis compared with other study groups.

Taken together, the present study displays a comprehensive array of molecular signatures after vaccination against typhoid fever and following typhoid challenge. The initial responses to both ViTT and ViPS were characterized by the activation of humoral immunity. Quantification of clonal expansion after vaccination demonstrates what we believe to be a novel correlate of protection against typhoid for ViTT recipients, while the identities of the clonotypes driving the upregulation were elucidated using a BCR clustering method. Molecular readouts from the post-challenge time points revealed that participants who received ViPS, and more so for those administered ViTT, showed reduced inflammation compared with the control vaccine recipients despite a typhoid diagnosis. These transcriptomic and immunological correlates can thus aid the understanding of the interplay between vaccines and typhoid infection.

## Methods

### Study design and participant sample collection.

Blood samples were obtained from participants enrolled in a randomized, phase IIb *S*. Typhi (Quailes strain) CHIM study (ClinicalTrials.gov NCT02324751) as described previously (2). Adult participants were consented to receive a single intramuscular dose of 1 of 3 vaccines: (a) ViPS (Typhim Vi, Sanofi Pasteur); (b) Vi-TT (Typbar-TCV, Bharat Biotech); or (c) a control meningococcal-ACWY conjugate vaccine (MENVEO, GlaxoSmithKline). Participants were orally challenged with 1 × 10^4^ to 5 × 10^4^ CFU *S*. Typhi bacteria around 4 weeks after vaccination and were monitored for 14 days. Participants were classified as TD when they had a fever of 38°C or higher for 12 hours or longer and/or had *S*. Typhi cultured from blood in the 14 days following challenge. Sequencing was carried out on whole blood samples collected at selected time points: for all participants, on the day of vaccination (V0), 1 day after vaccination (V1), 7 days after vaccination (V7), the day of challenge (D0), and 12 hours after challenge (D0+12h); for nTD participants, 7 days after challenge (D7) and 14 days after challenge (D14); and for TD participants, on the day of typhoid diagnosis (TD) (Figure 1 and Table 1). Formal statistical analyses were not conducted on the D14 time point because of insufficient sample numbers (*n* = 3). The variation of sample numbers across time points was due to (a) insufficient whole blood samples collected for RNA-Seq or (b) late typhoid diagnosis after the day-14 time point, in which case the participants did not have a TD visit per the protocol or undergo sample collection (Supplemental Table 1).

### RNA-Seq.

Whole blood RNA-Seq was performed using paired-end RNA-Seq. Peripheral blood was collected into Tempus RNA stabilization reagent (Life Technologies, Thermo Fisher Scientific). Total RNA was extracted from all samples using the Tempus Spin RNA Isolation kit (Life Technologies, Thermo Fisher Scientific). Quality control was carried out by measuring the RNA concentration of each library (mean RNA concentration, 109.56 ng/μL; median RNA concentration, 106.05 ng/μL; Supplemental Table 2). Briefly, libraries were prepared using a poly-A selection step to exclude ribosomal RNA species (read length: 75 bp paired-end, stranded), and samples were subsequently multiplexed and sequenced using an Illumina HiSeq sequencer (mean library size: 15,253,784 reads per sample; median library size: 15,029,616 reads per sample; Supplemental Table 2). Sequences assigned as ribosomal RNA, sex chromosome genes, mitochondrial RNA, or hemoglobin were excluded from the downstream analysis.

### Pre-processing RNA-Seq.

The sequencing data were aligned against the whole human (*Homo sapiens*) genome build GRCh38, using STAR (version 2.6.1d). Gene features were counted with HTSeq (version 0.9.1), using the human gene annotation general transfer format, version GRCh38.92 (www.ensembl.org). Genes with low counts across most libraries (genes without an abundance of greater than 1 count per million in 9 or more samples) were filtered out. rRNA, sex chromosome genes, mitochondrial RNA, and hemoglobin genes were excluded from the downstream analysis. HLA typing of RNA-Seq data using RNA2HLA (version 1) was performed to check the correct matching of samples collected from the same participants (84).

### Differential gene expression analysis.

Differential gene expression was undertaken using the R Bioconductor packages “edgeR” and “limma” (85–88). RNA-Seq data composition was normalized using the trimmed mean of M-value (TMM) method (85). Data were transformed using the limma “voom” function. A linear model was fitted to the data with the limma “lmFit” function using the empirical Bayes method (88). Paired analysis was conducted to compare pre-vaccination samples with each of the other study time points.

### Gene set and pathway analyses.

GSEA was conducted on gene lists ranked by the *t* statistic derived from the linear models comparing different study time points using the fgsea R package (89). We evaluated gene enrichment sets for the following Molecular Signatures Database (MSigDB) (http://www.gsea-msigdb.org/gsea/msigdb): C2 (curated gene sets), C5 (ontology gene), and C7 (immunological gene sets). Blood transcriptional module (BTM) analyses were also undertaken using the “tmod” R package for genes ranked by their log ratio (LR) value, and statistical testing for module expression was evaluated using the “tmodCERNOtest” function (90, 91). Weighted gene correlation network analysis (WGCNA) was used to describe modules of highly correlated genes within the RNA-Seq data (92). A step-by-step network construction and module detection approach was applied to batch-corrected, log_2_-transformed counts-per-million RNA-Seq data. A soft thresholding power of 9 was selected, and a signed-hybrid network was built applying the biweight midcorrelation as the adjacency function. A minimum module size was set to 20, and a dissimilarity threshold of 0.3 was used to merge highly similar modules.

### MaSigPro time-course analysis.

We performed a multiseries time-course analysis in order to identify differences in expression profiles between the 2 vaccination groups, using the R Bioconductor package MaSigPro (93). To assess the response to vaccination, time points V0, V1, V7, and D0 were selected, and the quadratic regression model was chosen (degree = 2), as only 1 intervention (vaccination) was predicted to influence gene expression, followed by resolution within this time series. Next, to assess the response to infection, D0, D0+12h, and TD time points were selected, and the linear regression model was used (degree = 1), since the overall perturbation in expression was expected to peak at TD, which was the last time point in the data set. The expression matrix containing the normalized (counts per million) expression values for each gene served as the main input. The regression fit was calculated for each gene, and a FDR below 0.05 was applied to determine the significant genes in each vaccine group. Next, to identify significant differences in the profiles between the vaccine groups the backward (step.method=“backward”) variable selection procedure was used with a *P* value cutoff of 0.05. Finally, cluster analysis (hclust) was performed to group the genes from the list of significant genes according to similar profiles. The optimal number of clusters was determined by Mclust (k.mclust=TRUE). The clusters were visualized using the see.genes function, with regression fit curves plotted for each vaccination group. Only participants who were not diagnosed with typhoid fever after challenge (nTD) from both vaccination groups were included in the post-vaccination analysis. Only those participants who were diagnosed with typhoid fever (TD) were included in the post-challenge analysis.

### Immunome data preparation.

Immunome data were extracted from the transcriptome dataset using t he MiXCR functions “Align,” “Assemble partial,” “Assemble,” “Export clones min,” and “Export clones” — all under default settings (94, 95). Extracted Immunome data were reorganized to achieve a format suitable for BCR analysis through conversion using VDJtools under default settings (96).

### IGHV usage analysis.

VDJtools outputs for BCR information at every data point were combined by vaccine type and challenge outcome. The percentage of IGHV usage in the total BCR repertoire per time point across participants of different challenge outcomes was calculated, and plots were generated using ggplot2 (97).

### Clonal expansion analysis.

Clonal expansion size classification and visualization were performed using the Immunarch function “repClonality” under the “homeo” setting (15). The “homeo” setting calculates the relative abundances of clonal groups as a proportion of the repertoire. For expansion analysis, BCR clonotypes utilizing *IGHV3-23* were compared with likely TT-binding BCR clonotypes. TT-binding BCR clonotypes were identified from past literature (20–29). Likely TT-binding BCRs were defined by the BCR clonotypes identified in the study, which comprises a CDR H3 sequence differing by at most 1 amino acid (1 Hamming distance) from the CDR H3 sequences of TT-binding BCRs, while having the same CDR H3 length. Total clone counts, defined by the sum of each unique B cell clonotype identified from MiXCR multiplied by the total number of copies expressed, were calculated for (a) all B cell clonotypes comprising IGHV3-23 and (b) all likely TT-binding B cell clonotypes. The ratio was calculated by dividing (a) by (b) for each participant at V7 who received ViTT.

### BCR CDR H3 sequence clustering.

To classify and cluster biologically similar BCRs that possess specificities similar to those of an antigen as single entities for downstream analysis, we retrieved individual clonotype information using VDJtools followed by clustering using a BCR clustering iteration method. The Hamming distance of all CDR H3 sequences was calculated in a matrix. A randomly selected BCR was allocated a cluster number of 1, acting as a “seed” sequence. Any sequences within 1 Hamming distance of this “seed” sequence was allocated the same cluster number, forming the second layer of sequences. These sequences at the second layer acted as additional “seed” sequences, whereby additional sequences within 1 Hamming distance were assigned to the same cluster. All CDR H3 sequences were allocated a cluster number through iteration of this process. With different clustering runs, different cluster numbers may have been assigned to the same cluster because of random cluster number allocation. Visualization of cluster networks was performed using the iGraph R package (98).

### Cluster sequence conservation analysis.

Logo and pLogo plots for clusters identified through CDR H3 sequence clustering were produced using the ggseqlogo package in R and the pLogo webtool, respectively (99, 100). pLogo ran with the replicates retained. The foreground of the pLogo plots constitutes all CDR H3 sequences within the CDR H3 sequence cluster. The background contains all other CDR H3 sequences derived from B cell clonotypes with the same IGHV usage and are of the same CDR H3 length as the foreground. Amino acid sequence alignment was performed with Clustal Omega (19).

### Statistics.

Statistical analyses were performed in R (version 4.0.5). Statistical significances were calculated for differential gene expression analysis readouts using the R Bioconductor packages edgeR and limma. Statistical significance for the repertoire analyses was calculated using the R package ggpubr and the built-in functions “t.test” and “wilcox.test” for 2-tailed *t* tests and Mann-Whitney *U* tests, respectively. Statistical analyses for the time series gene profiles were carried out using the R Bioconductor package MaSigPro. Benjamini-Hochberg–adjusted *P* values of less than 0.05 were considered statistically significant unless specified otherwise in Methods.

### Study approval.

Samples included in this study were collected from a phase IIb *S*. Typhi (Quailes strain) CHIM study (ClinicalTrials.gov: NCT02324751) performed at the Centre for Clinical Vaccinology and Tropical Medicine, Churchill Hospital, Oxford, United Kingdom. Approval of the study protocol was granted by the sponsor (University of Oxford), the South Central Oxford A Ethics Committee (14/SC/1427), and the Medicines and Healthcare Products Regulatory Agency (Eudract 2014-002978-36).

### Data and materials availability.

Transcriptomics data are available in the NCBI’s Gene Expression Omnibus (GEO) database (GEO GSE217667). Code used for BCR clustering is available on GitHub (https://github.com/HendersonZhu/BCR_TCR_clustering/commits/main; commit ID: 65c0076d4155c6ccf66cc2896491cb1cbcefbddc). Values for all data points in the figures can be found in the supplemental supporting data values file.

## Author contributions

DO, IC, CJ, MMG, CJB, and AJP conceptualized the study, and DO supervised the study. DLC, LB, EJ, and JH performed the laboratory work. HZ, IC, and DO performed the in silico analysis. JT and DFK reviewed and improved the in silico analysis. AJP acquired funding. HZ, IC, and DO wrote the original draft of the manuscript. All authors critically reviewed and approved the final version of the manuscript. All authors had full access to all the data in the study and had final responsibility for the decision to submit for publication. HZ and IC are listed as co–first authors on the basis of their equal contributions to the work.

## Supplementary Material

Supplemental data

Supporting data values

## Figures and Tables

**Table 1:**
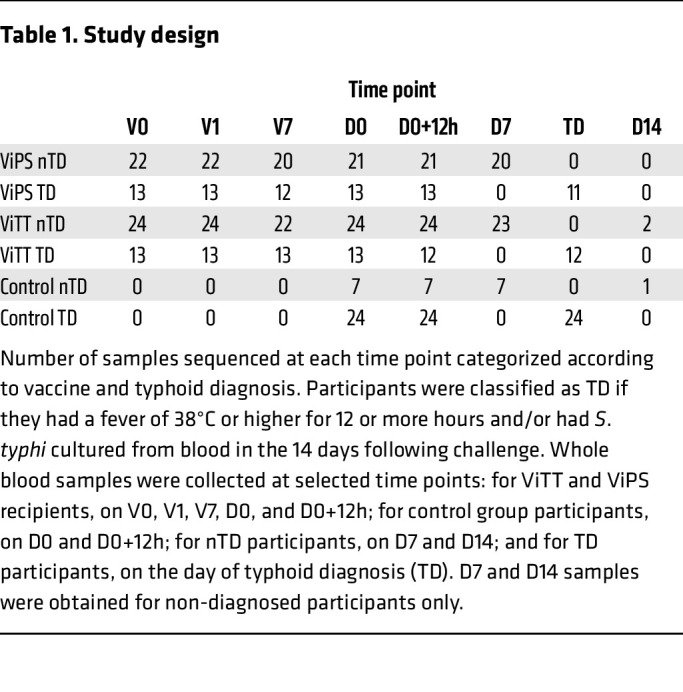
Study design

## Version 1 07/04/2023

In-Press Preview

## Version 2 08/15/2023

Electronic publication
