# Supplementary material for: Molecular correlates of vaccine-induced protection against typhoid fever
Source: J Clin Invest. 2023 Aug 15;133(16):e169676. doi: 10.1172/JCI169676 (PMC10425215; doi:10.1172/JCI169676)
Supplement: Supplemental data [file jci-133-169676-s084.pdf]

Supplemental information for “Molecular correlates of vaccine-induced protection against typhoid fever”

Supplementary information

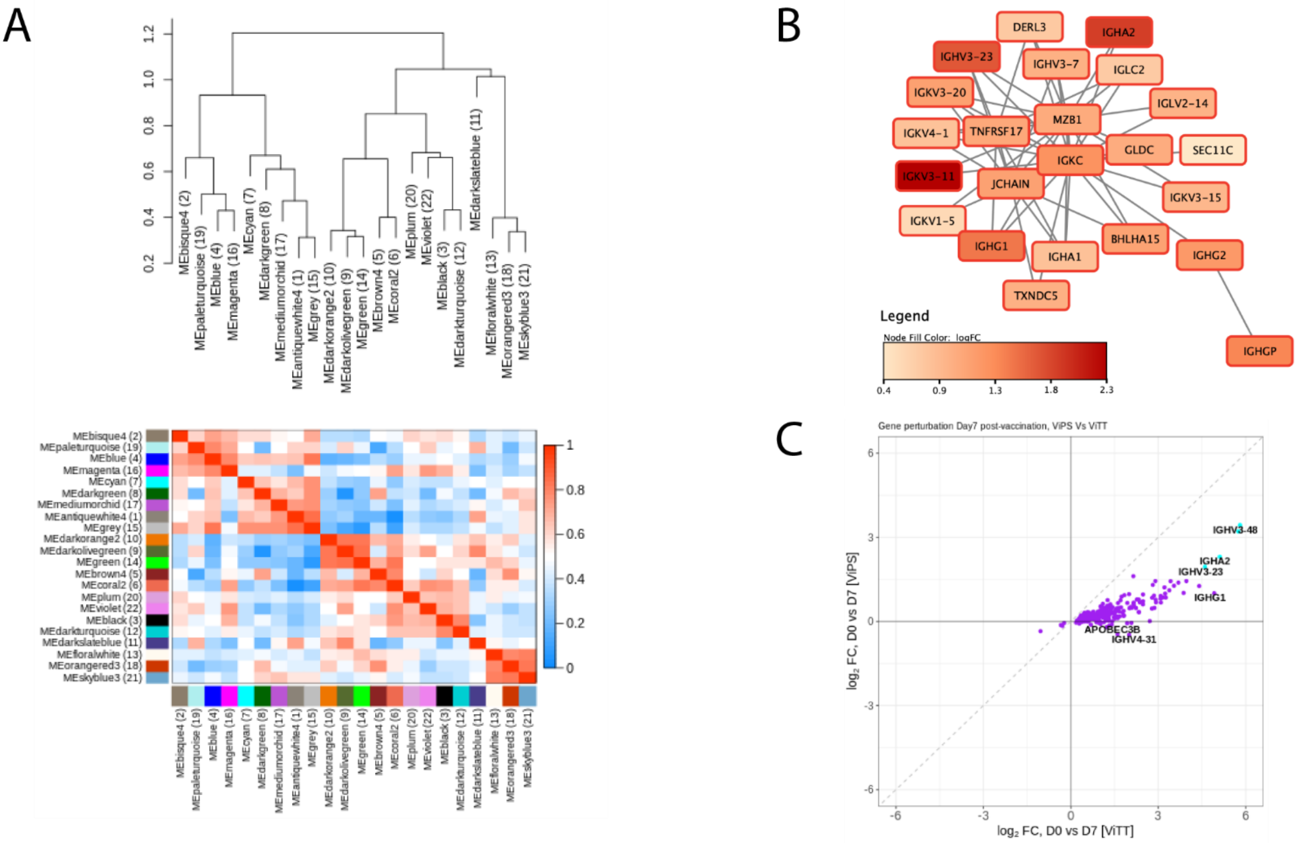

Supplementary figure 1: **Gene module analysis seven-days post-vaccination compared with baseline.** (A) Weighted gene correlation network analysis (WGCNA) module correlation matrix. (B) Gene within the skyblue3 module with the highest intramodular connectivity. (C) Agreement plot of fold-change of differentially expressed genes 7 days ($p < 0.05$) after ViPS (y-axis) or ViTT (x-axis) compared with pre-vaccination (purple are gene differentially expressed after ViTT only, cyan genes are differentially expressed after either vaccine).

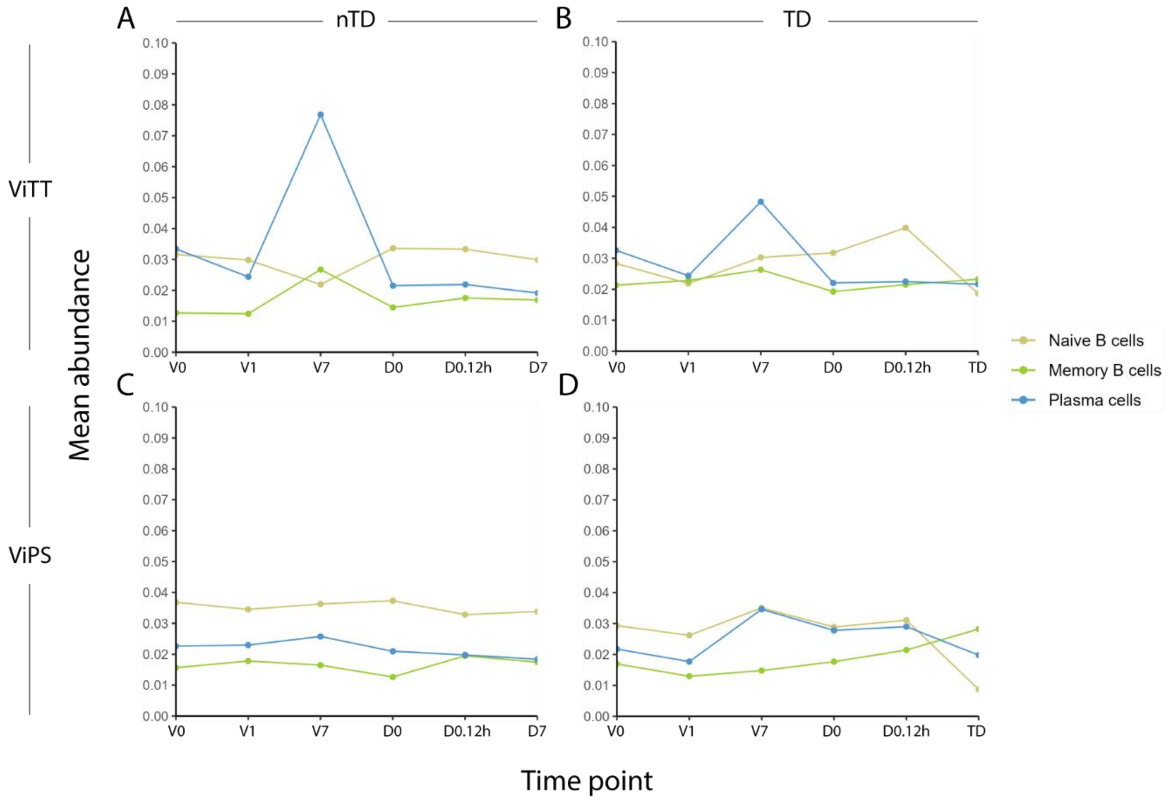

Supplementary figure 2: **Mean abundance as outputted from CybersortX of naïve B cells, memory B cells, and plasma cells across study time points.** For (A) ViTT nTD participants; (B) ViTT TD particiapants; (C) ViPS nTD participants; (D) ViPS TD participants.

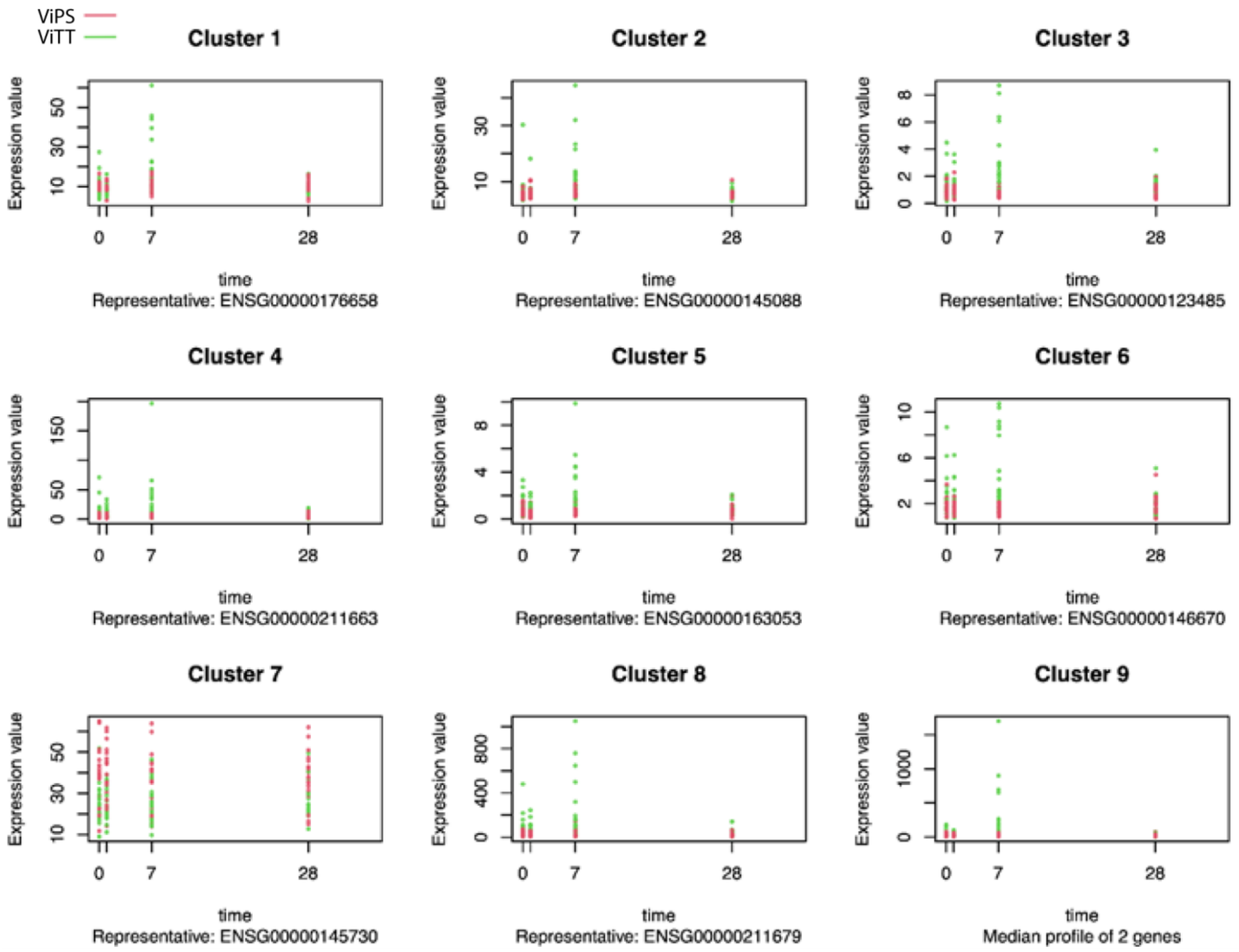

Supplementary figure 3: **Significantly different expression profiles across the time points following vaccination among nTD participants.** Representative example gene from each cluster is plotted. Regression fit curves are shown for each vaccine group, ViPS recipients are indicated in red, and ViTT in green.

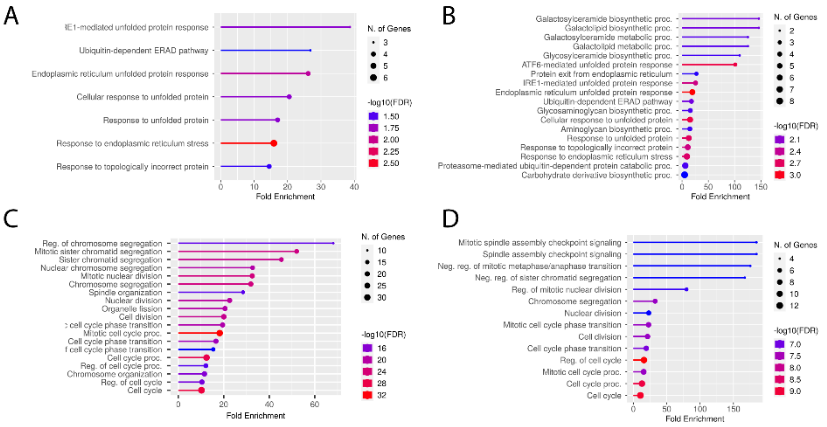

Supplementary figure 4: **Significantly enriched GO-terms (biological process) in each of the big clusters containing the genes with unique profile in ViTT vaccinated participants post vaccination.** Up to 20 pathways are shown, FDR cut-off 0.05. (A) Cluster 1; (B) Cluster 2; (C) Cluster 3; (D) Cluster 6.

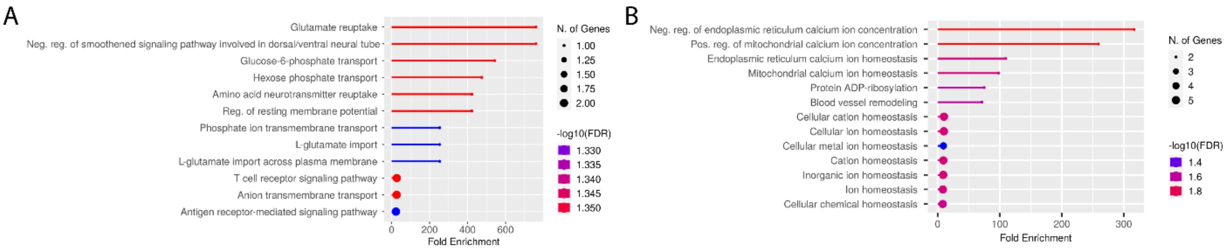

Supplementary figure 5: **Significantly enriched GO-terms (biological process) in each of the big clusters containing the genes with unique profile in ViPS vaccinated participants post vaccination.** Only clusters with >10 genes were included, up to 20 pathways are shown, FDR cut-off 0.05. Cluster 3 did not have any GO-terms enriched under the defined cut-off. (A) Cluster 4; (B) Cluster 5.

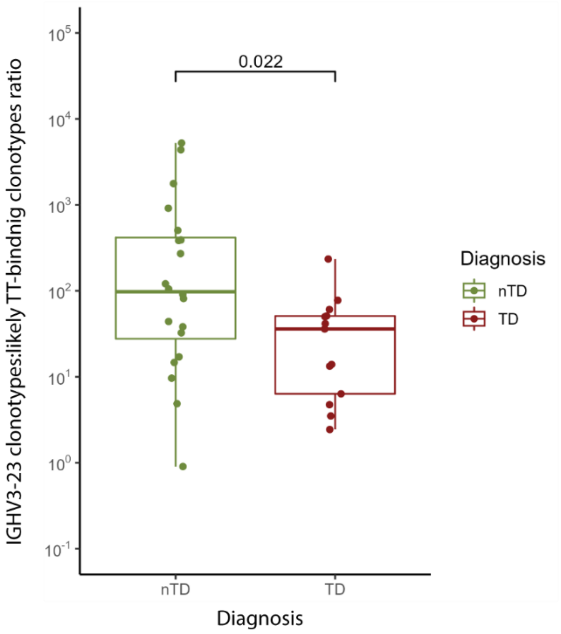

Supplementary figure 6**: Ratio of clonotypes using IGHV3-23 to likely TT-binding clonotypes in ViTT participants at seven-days post-vaccination (V7).** Significance was determined by Mann–Whitney U test.

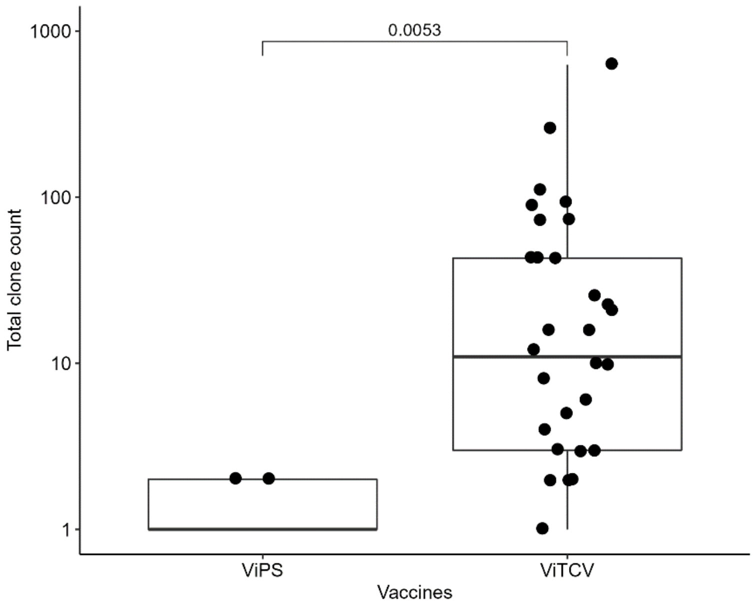

***Supplementary figure 7*: Total clone count of likely TT-binding BCR clonotypes for ViPS and ViTT recipients at seven-days post-vaccination (V7).** Significance was determined by Mann–Whitney *U* test.

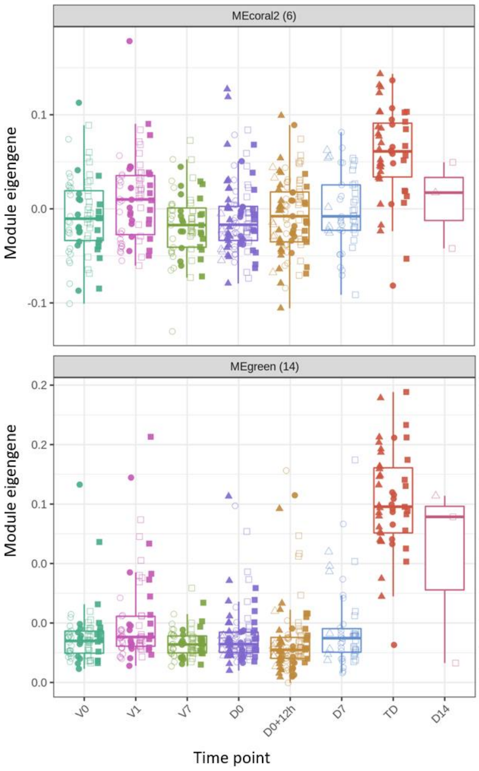

module	size	p-val	Bonf	nlnTerm	ont	term name
coral2	752	1.6e-30	2.9e-26	209	BP	immune response
coral2	752	1.1e-29	2e-25	114	BP	myeloid leukocyte activation
coral2	752	8.4e-29	1.5e-24	102	BP	leukocyte degranulation
coral2	752	1.8e-27	3.2e-23	115	BP	leukocyte activation involved in immune
coral2	752	2.8e-27	5.1e-23	115	BP	cell activation involved in immune respo
coral2	752	6.9e-27	1.3e-22	95	BP	neutrophil activation involved in immune
coral2	752	8.1e-27	1.5e-22	96	BP	neutrophil activation
coral2	752	1.8e-26	3.2e-22	94	BP	neutrophil degranulation
coral2	752	2.5e-26	4.5e-22	95	BP	neutrophil mediated immunity
coral2	752	3.3e-26	6.1e-22	259	BP	immune system process

module	size	p-val	Bonf	nlnTerm	ont	term name
green	385	6.4e-42	1.2e-37	123	BP	response to external biotic stimulus
green	385	6.4e-42	1.2e-37	123	BP	response to other organism
green	385	1.6e-38	2.9e-34	104	BP	defense response to other organism
green	385	6.3e-36	1.2e-31	122	BP	defense response
green	385	5.1e-35	9.3e-31	90	BP	innate immune response
green	385	1.3e-34	2.4e-30	54	BP	defense response to virus
green	385	2e-32	3.7e-28	58	BP	response to virus
green	385	9.7e-28	1.8e-23	130	BP	immune response
green	385	1.2e-26	2.2e-22	94	BP	response to cytokine
green	385	2e-23	3.6e-19	37	BP	response to interferon-gamma

Supplementary figure 8: **Weighted gene correlation network analysis (WGCNA) module and top 10 enriched gene ontologies.**

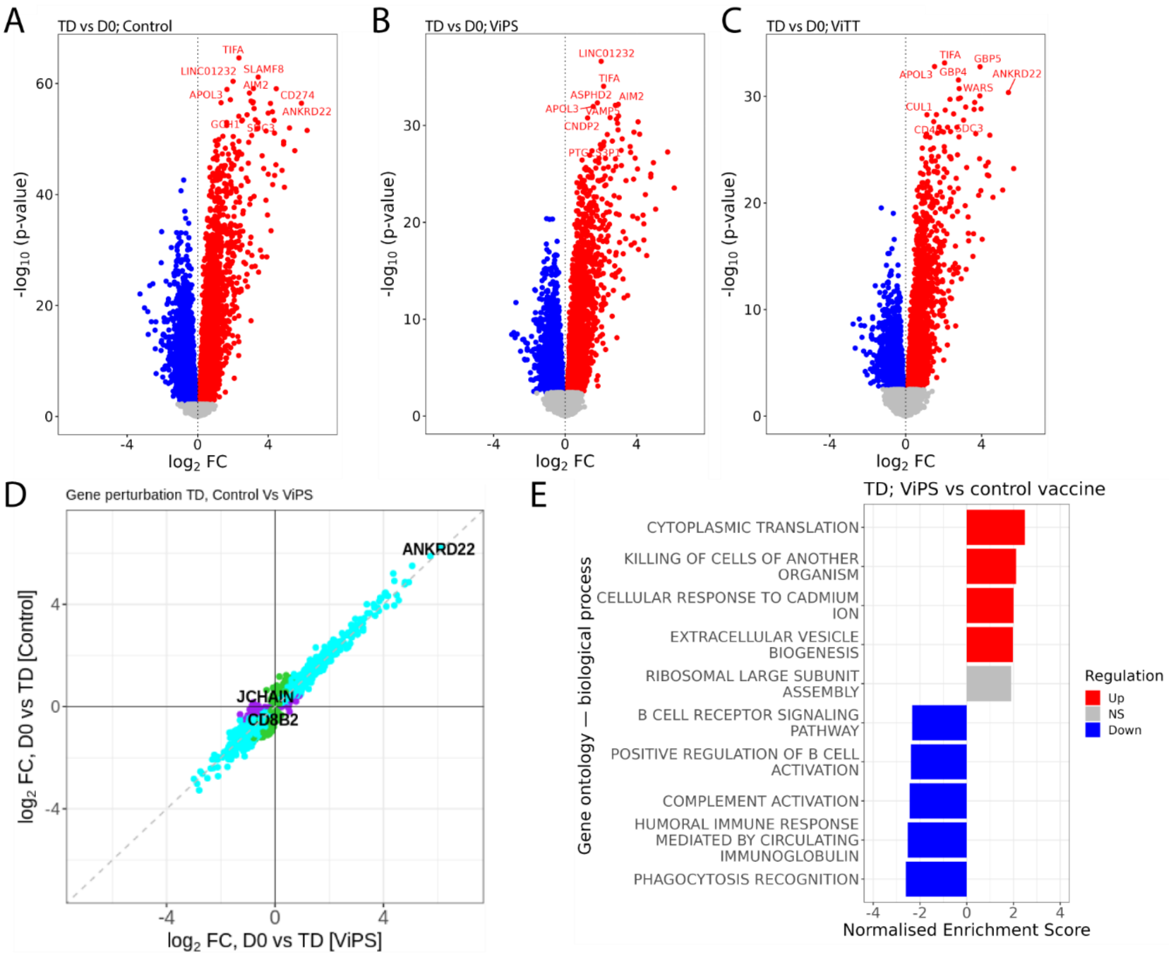

***Supplementary figure 9:* Blood gene expression profile at typhoid diagnosis (TD).** (A) Volcano plot of differences in gene expression at the diagnosis time point in the control vaccine group compared with the day of challenge. (B) Volcano plot of differences in gene expression at the diagnosis time point in the ViPS vaccine group compared with the day of challenge. (C) Volcano plot of differences in gene expression at the diagnosis time point in the ViTT vaccine group compared with the day of challenge. (D) Agreement plot of changes in gene expression (differentially expressed genes only) at typhoid diagnosis in those who received ViTT compared with control vaccine recipients. (E) The top 5 upregulated and downregulated pathways from gene set enrichment analysis (GSEA) at the day of typhoid diagnosis in ViTT compared with control vaccine recipients.

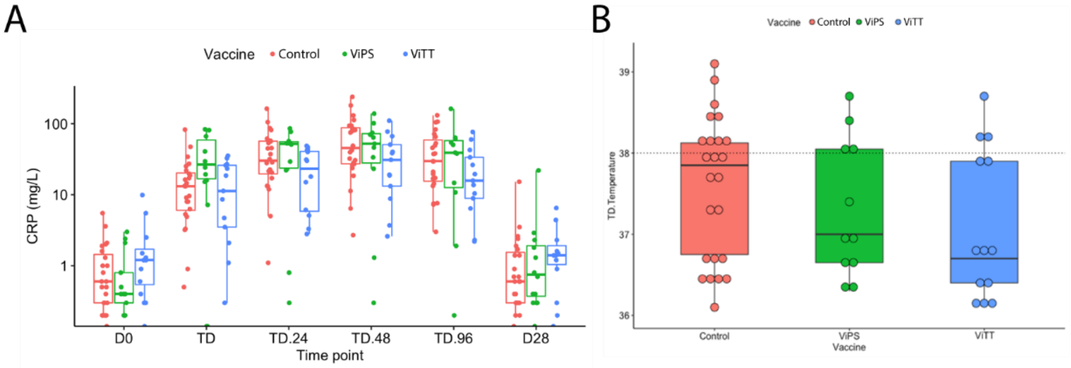

Supplementary figure 10: **Clinical features around typhoid diagnosis (TD).** (A) C-reactive protein measured in blood of infected participants across the time course of typhoid infection. Median concentration in each group is shown in coloured lines. D28 denotes 28 days post-challenge. (B) temperature measured at time point of diagnosis. Dotted line corresponds to the 38°C as a definition of fever.

Supplementary table 1: **Information of participants unable to have RNA-Seq data collected.**

Vaccine	Time point without RNA-Seq data	Diagnosis	Reason of absence
ViPS	D7	nTD	Blood sample for serological analyses collected, however the tube for RNA-Seq was not collected.
ViPS	TD	TD	Late typhoid diagnosis post-D14 time point, therefore these participants did not have a TD visit by the protocol and the samples were not collected.
ViPS	D7	nTD	Blood sample for serological analyses collected, however the tube for RNA-Seq was not collected.
ViPS	TD	TD	Late typhoid diagnosis post-D14 time point, therefore these participants did not have a TD visit by the protocol and the samples were not collected.
ViPS	D0	nTD	RNA extraction failed as there was not sufficient RNA for sequencing.

Supplementary table 2: **Library information.**

**Library name**	**Vaccine**	**Time point**	**Diagnosis**	**RNA concentration (ng/µl)**	**Library size (reads)**
VAST6958375	ViPS	D0+12h	nTD	209	14939614
VAST6958376	ViPS	V0	nTD	131.4	11900579
VAST6958377	ViPS	D0+12h	nTD	198.6	14379284
VAST6958378	ViPS	V1	nTD	40.4	15260735
VAST6958379	ViPS	D7	nTD	121.2	15192248
VAST6958380	ViPS	V7	nTD	96.2	14323921
VAST6958381	ViPS	V0	nTD	133	12524782
VAST6958382	ViPS	V1	nTD	176.6	13740518
VAST6958383	ViPS	D0	nTD	146.6	11376469
VAST6958384	ViPS	V7	nTD	118.4	12632335
VAST6958385	ViPS	D7	nTD	193.7	10036531
VAST6958386	ViPS	D0	nTD	125.7	12529480
VAST6958387	ViPS	V0	TD	137.5	12642530
VAST6958388	Control	D0+12h	nTD	177	10397985
VAST6958389	ViPS	V1	TD	168.6	12993467
VAST6958390	ViPS	D0+12h	TD	154.3	13822251
VAST6958391	ViTT	D0+12h	TD	229.7	10326294
VAST6958392	ViTT	V0	TD	77	13089702
VAST6958393	Control	D0+12h	TD	180.1	14883572
VAST6958394	Control	D0	TD	71.7	15284882
VAST6958395	Control	D7	nTD	143.1	12991247
VAST6958396	ViPS	V7	TD	47.1	16533329
VAST6958397	ViTT	V1	TD	139.1	9780590
VAST6958398	ViTT	V7	TD	127.8	14428961
VAST6958399	Control	D0	nTD	44.4	9289968
VAST6958400	ViPS	D0	TD	169.5	12200827
VAST6958401	ViTT	D0	TD	120.6	10392794
VAST6958402	ViTT	V0	nTD	105.9	13733456
VAST6958403	ViPS	V0	TD	144.3	14854687
VAST6958404	ViTT	V1	nTD	143.4	13225149
VAST6958405	ViPS	V1	TD	103.2	12859594
VAST6958406	ViTT	TD	TD	93.1	10376667
VAST6958407	ViPS	TD	TD	126.5	12567377
VAST6958408	ViTT	V7	nTD	148	12682239
VAST6958409	ViPS	V7	TD	83.1	12440722
VAST6958410	Control	TD	TD	89.4	14131423
VAST6958411	ViPS	V0	nTD	147.3	12410330
VAST6958412	ViTT	V0	TD	106.6	15270502
VAST6958413	ViPS	V0	nTD	184.1	13274413
VAST6958414	ViTT	D0+12h	nTD	129.2	11996942
VAST6958415	ViPS	V1	nTD	181.7	11747961
VAST6958416	ViPS	D0+12h	TD	156.9	11848743
VAST6958417	ViTT	V1	TD	141.5	13002169
VAST6958418	Control	D0+12h	TD	137.9	12296075
VAST6958419	ViPS	V1	nTD	171.3	11835664

VAST6958420	ViTT	V1	nTD	127.5	15682208
VAST6958421	ViTT	D0	nTD	80	14123857
VAST6958422	ViTT	D7	nTD	109	15977686
VAST6958423	ViPS	V7	nTD	166.3	12138133
VAST6958424	ViPS	V7	nTD	199.4	11214784
VAST6958425	ViTT	V7	nTD	73.7	14336415
VAST6958426	ViTT	V7	TD	90.9	12375168
VAST6958427	Control	D0	TD	71.6	12275728
VAST6958428	ViPS	D0	TD	169.8	11234082
VAST6958429	ViTT	D14	nTD	102.3	15258013
VAST6958430	ViPS	V0	nTD	95.7	11216787
VAST6958431	ViTT	V0	nTD	23.2	15861762
VAST6958432	Control	TD	TD	98.6	14028303
VAST6958433	ViTT	V0	nTD	88.6	19048374
VAST6958434	ViTT	V1	nTD	95.5	13941802
VAST6958435	ViTT	V1	nTD	110.1	18888318
VAST6958436	ViPS	V1	nTD	201.3	11489064
VAST6958437	ViPS	TD	TD	106.8	13662745
VAST6958438	ViPS	V7	nTD	92.2	11098830
VAST6958439	ViTT	V0	nTD	60.6	12998666
VAST6958440	ViPS	D0+12h	nTD	186.7	13159347
VAST6958441	ViTT	V7	nTD	75.7	14742272
VAST6958442	ViTT	V7	nTD	125.1	14707589
VAST6958443	ViTT	D0	TD	128.9	15027803
VAST6958444	ViTT	V0	nTD	94.5	14360036
VAST6958445	ViTT	D0	nTD	74.1	17461812
VAST6958446	ViPS	D0	nTD	113.4	13151675
VAST6958447	ViTT	D0+12h	TD	132.9	17445309
VAST6958448	Control	D0	TD	92.3	16624555
VAST6958449	ViPS	D0+12h	nTD	159.7	17374277
VAST6958450	ViPS	D0	nTD	159.1	12809515
VAST6958451	Control	D0+12h	TD	117.2	18333112
VAST6958452	ViPS	V0	nTD	89.2	16796123
VAST6958453	ViTT	D0+12h	nTD	92.7	17597972
VAST6958454	ViTT	V0	TD	104.8	18570799
VAST6958455	ViTT	V1	nTD	66.1	15286890
VAST6958456	ViPS	V1	nTD	74.7	16999399
VAST6958457	ViTT	V1	TD	106.2	14457597
VAST6958458	Control	TD	TD	97.9	16878158
VAST6958459	ViTT	V7	TD	117.1	16534259
VAST6958460	ViTT	D0	nTD	142.5	15373798
VAST6958461	ViPS	D0	nTD	130.2	12133249
VAST6958462	ViTT	D0	nTD	111.7	15398718
VAST6958463	ViPS	V7	nTD	85.1	13662591
VAST6958464	ViTT	V7	nTD	79.2	11352750
VAST6958465	ViTT	TD	TD	78.3	15986583
VAST6958466	ViTT	V0	nTD	142.7	14181423
VAST6958467	ViPS	D7	nTD	130.6	12361997

VAST6958468	ViPS	D7	nTD	90.9	13868645
VAST6958469	ViPS	V0	nTD	80.8	13625230
VAST6958470	ViTT	D7	nTD	72.1	14361650
VAST6958471	ViTT	V0	nTD	146.2	16200495
VAST6958472	ViPS	V1	nTD	73.9	14553290
VAST6958473	ViTT	V1	nTD	124.1	16857315
VAST6958474	ViTT	V1	nTD	128	17125209
VAST6958475	ViPS	V7	nTD	67.5	17937073
VAST6958476	ViTT	D0+12h	nTD	151.9	12626710
VAST6958477	ViTT	V7	nTD	121.5	14416184
VAST6958478	ViTT	V7	nTD	92.7	16934978
VAST6958479	ViPS	D0+12h	nTD	175	11422789
VAST6958480	Control	D0	nTD	79.5	17315791
VAST6958481	ViTT	D0	TD	75.5	17021350
VAST6958482	ViTT	D0	nTD	69.6	14704983
VAST6958483	ViTT	D0+12h	nTD	148.1	13522447
VAST6958484	ViPS	V0	TD	132.4	16975497
VAST6958485	ViTT	V0	TD	63.1	17870395
VAST6958486	ViPS	V0	nTD	73.5	19008832
VAST6958487	ViPS	V0	TD	90.2	18306331
VAST6958488	ViPS	V1	TD	135.7	18725297
VAST6958489	ViPS	V1	nTD	68.5	14753257
VAST6958490	ViTT	D7	nTD	132.8	14587548
VAST6958491	ViPS	D7	nTD	121.1	16646356
VAST6958492	ViTT	V1	TD	91.8	19337439
VAST6958493	ViTT	D7	nTD	82.3	13386011
VAST6958494	ViPS	V1	TD	140.4	16919551
VAST6958495	ViPS	V7	TD	143.9	13746105
VAST6958496	ViTT	V7	TD	57.1	14742914
VAST6958497	ViPS	V7	nTD	80.9	14274168
VAST6958498	ViPS	D0+12h	nTD	93.1	18679168
VAST6958499	ViPS	V0	nTD	108.3	13413969
VAST6958500	ViTT	D0+12h	nTD	99	17758727
VAST6958501	ViPS	V7	TD	157.4	13987243
VAST6958502	Control	D0+12h	nTD	203.9	14864894
VAST6958503	ViTT	D0+12h	TD	140.8	14688884
VAST6958504	ViTT	D0	nTD	118.7	13243704
VAST6958505	ViPS	D0	nTD	73.7	17074037
VAST6958506	ViTT	D0	nTD	153.2	15031428
VAST6958507	Control	D0	TD	15.5	15832173
VAST6958508	ViTT	V0	TD	111.2	11301872
VAST6958509	ViPS	V1	nTD	150.7	12714404
VAST6958510	ViTT	V1	TD	124.9	14069973
VAST6958511	ViPS	V7	nTD	228.8	14210342
VAST6958512	ViTT	V7	TD	149.4	9668431
VAST6958513	Control	D0	nTD	90.8	10475623
VAST6958514	ViTT	V0	nTD	87.6	17075459
VAST6958515	ViPS	D0	nTD	98.1	13429001

VAST6958516	ViTT	D0	TD	90.3	13454058
VAST6958517	Control	D0	TD	60.5	13550503
VAST6958518	ViPS	D0	nTD	71.8	15518415
VAST6958519	Control	D0	TD	124.4	13814626
VAST6958520	Control	D0	TD	103.7	11180332
VAST6958521	ViPS	D0	TD	154.4	14289879
VAST6958522	Control	D7	nTD	109.2	13995279
VAST6958523	ViPS	D0	TD	107.7	15659812
VAST6958524	ViTT	V0	nTD	48.3	14101571
VAST6958525	ViTT	D7	nTD	20.2	16204016
VAST6958526	ViTT	V1	nTD	79.9	14530550
VAST6958527	ViPS	D7	nTD	82.7	12595983
VAST6958528	ViTT	V1	TD	68.7	12368936
VAST6958529	Control	D14	nTD	78.7	18568907
VAST6958530	ViTT	V0	TD	62.3	22216975
VAST6958531	ViTT	V0	nTD	97.5	17104892
VAST6958532	ViTT	V0	nTD	57.8	16860886
VAST6958533	ViTT	V1	nTD	138.9	13238786
VAST6958534	ViTT	TD	TD	56	15543112
VAST6958535	ViTT	V0	nTD	60.1	14292645
VAST6958536	ViTT	V1	nTD	160.9	14360918
VAST6958537	ViTT	V1	nTD	105.8	11648071
VAST6958538	Control	D0+12h	TD	102.1	16025282
VAST6958539	ViTT	V7	nTD	87.2	13260498
VAST6958540	ViTT	D0+12h	nTD	132.9	13710386
VAST6958541	ViTT	V1	nTD	73.9	16365432
VAST6958542	ViPS	D0+12h	nTD	95.6	16176432
VAST6958543	ViTT	D0+12h	nTD	105.1	12984292
VAST6958544	ViTT	V7	nTD	105.5	15620919
VAST6958545	ViTT	V7	nTD	173.1	12474265
VAST6958546	ViTT	V0	TD	105	15053881
VAST6958547	ViTT	V7	TD	114.7	13780469
VAST6958548	ViTT	V7	nTD	91.4	13957019
VAST6958549	ViTT	V7	nTD	116.4	13307519
VAST6958550	Control	D0	TD	115.2	18520430
VAST6958551	ViPS	D0	nTD	86.7	13389941
VAST6958552	ViTT	D0	TD	76.3	11533754
VAST6958554	Control	TD	TD	58.8	14724516
VAST6958556	ViTT	V0	TD	68.9	18499850
VAST6958557	ViPS	V0	nTD	32.2	16482829
VAST6958558	ViPS	V1	nTD	51.8	17224818
VAST6958559	ViTT	V1	TD	107.8	13762768
VAST6958560	ViTT	V0	TD	112.9	8283869
VAST6958561	ViPS	D7	nTD	16.3	12258270
VAST6958562	ViTT	V0	nTD	70.6	9543163
VAST6958563	ViTT	D7	nTD	102.6	11326002
VAST6958564	ViTT	V0	nTD	97.9	10525631
VAST6958565	ViTT	D7	nTD	128.6	11635212

VAST6958566	ViTT	V1	TD	41.2	20008141
VAST6958567	ViTT	V1	nTD	136.8	11697083
VAST6958568	ViPS	D0+12h	nTD	48.4	13079431
VAST6958569	ViTT	V1	nTD	39.9	13559660
VAST6958570	ViTT	V1	TD	25.5	14346219
VAST6958571	ViTT	D0	nTD	49.8	19666381
VAST6958572	ViTT	D0+12h	TD	51.6	12060350
VAST6958573	ViTT	V7	TD	32.9	15534524
VAST6958574	Control	D0+12h	TD	95.8	15507785
VAST6958575	ViTT	D0	nTD	21.3	12776487
VAST6958576	Control	D0+12h	TD	22.1	14973246
VAST6958577	ViTT	D0	nTD	11.1	12909551
VAST6958578	Control	D0+12h	nTD	9.2	13823826
VAST6958579	ViTT	D0	nTD	100.1	18868397
VAST6958580	ViTT	D0	TD	79.8	14076008
VAST6958581	ViPS	V7	nTD	50.7	15880266
VAST6958582	ViTT	D0	nTD	54.6	20692713
VAST6958584	ViPS	D0+12h	TD	158.5	16499369
VAST6958585	ViTT	V7	TD	155.6	16243275
VAST6958586	ViPS	D0+12h	TD	118.6	15040064
VAST6958587	ViTT	V7	TD	121.2	14146176
VAST6958588	Control	D0+12h	TD	204.7	17297186
VAST6958589	ViTT	V1	nTD	114.6	15875934
VAST6958590	ViTT	V7	nTD	116.5	17748147
VAST6958591	ViTT	V0	nTD	104.5	16668873
VAST6958593	ViTT	V0	TD	74.5	14597457
VAST6958594	ViTT	V0	nTD	90.9	14831235
VAST6958595	ViTT	V0	nTD	71.5	16197934
VAST6958597	ViTT	V1	TD	74.9	11650275
VAST6958598	ViPS	V1	nTD	49.7	14864010
VAST6958599	ViTT	V1	nTD	76.2	11920132
VAST6958600	ViTT	V1	nTD	143.2	13194164
VAST6958601	ViPS	V0	nTD	101.7	17057212
VAST6958602	ViTT	V0	nTD	53.2	16116851
VAST6958603	ViTT	V0	TD	68.4	18993185
VAST6958604	ViTT	V1	nTD	105.6	14028494
VAST6958605	ViTT	V1	TD	52.1	16417245
VAST6958606	ViTT	V7	TD	133.6	10809650
VAST6958607	Control	D7	nTD	59.1	16006710
VAST6958608	ViTT	V7	nTD	36.8	13856580
VAST6958609	ViTT	D0	TD	148.1	15854243
VAST6958610	ViTT	V7	nTD	124.4	11914529
VAST6958611	ViPS	D7	nTD	87.3	14555681
VAST6958612	ViTT	V7	TD	83.3	13323133
VAST6958613	ViTT	V7	nTD	144.4	15964928
VAST6958614	ViPS	V7	nTD	62.5	25021027
VAST6958615	ViTT	V0	nTD	85.2	15469472
VAST6958616	Control	TD	TD	116.7	14937365

VAST6958617	ViTT	V0	TD	82.3	17664824
VAST6958618	ViPS	V0	nTD	58.4	16376999
VAST6958619	ViPS	TD	TD	82.8	18052304
VAST6958620	ViPS	V1	nTD	71.9	21231279
VAST6958621	ViTT	V0	TD	135.7	16968508
VAST6958622	ViPS	D0	nTD	42.3	16731252
VAST6958623	ViTT	V1	TD	97	17576828
VAST6958624	ViTT	D0	TD	75.7	15606884
VAST6958625	ViTT	V0	nTD	81	15545728
VAST6958626	ViTT	V0	nTD	95.9	14531315
VAST6958627	ViTT	V1	nTD	106.3	20453385
VAST6958628	ViTT	V0	nTD	76.6	18401525
VAST6958629	ViTT	V0	nTD	209.4	13364163
VAST6958630	Control	TD	TD	67	14110974
VAST6958631	ViTT	V1	nTD	140.5	16788244
VAST6958632	ViTT	V0	nTD	150.4	16233186
VAST6958633	ViTT	D0	nTD	167.5	13079375
VAST6958634	ViTT	D0	nTD	97.1	17463848
VAST6958635	ViPS	TD	TD	102.3	19606220
VAST6958636	ViTT	D0	TD	126.4	16351375
VAST6958637	ViTT	V1	nTD	104.8	17423283
VAST6958638	ViTT	V1	nTD	68.9	17000396
VAST6958639	ViTT	V1	nTD	45.7	17017774
VAST6958640	Control	TD	TD	112.3	16142831
VAST6958642	ViTT	V1	TD	90.9	19822553
VAST6958643	ViTT	D0+12h	TD	87.3	14378918
VAST6958644	ViTT	V1	nTD	95.5	13313301
VAST6958645	ViPS	D0+12h	nTD	108.9	16678729
VAST6958646	Control	D0+12h	TD	131.3	17561223
VAST6958647	Control	TD	TD	77.1	14745323
VAST6958648	ViTT	V7	nTD	106.6	20595694
VAST6958649	ViTT	V7	TD	56.9	14606029
VAST6958650	ViTT	V7	nTD	89.9	18025141
VAST6958651	ViTT	V7	nTD	153.8	17001773
VAST6958652	ViTT	V7	nTD	103.8	13943847
VAST6958653	ViTT	TD	TD	133.2	13294846
VAST6958654	ViTT	V7	nTD	124	13941852
VAST6958655	ViPS	D7	nTD	169.4	11439263
VAST6958656	ViTT	V7	nTD	127	12242282
VAST6958657	ViTT	V7	TD	261.7	12532245
VAST6958658	ViTT	D0+12h	nTD	209.5	13791671
VAST6958659	ViPS	V0	nTD	149.7	17779008
VAST6958660	ViTT	D0+12h	nTD	123.5	13249418
VAST6958661	ViPS	V1	nTD	147.9	12580076
VAST6958662	ViTT	D0+12h	nTD	159	17312716
VAST6958663	ViTT	D0	nTD	146.9	13475499
VAST6958664	ViTT	D0	nTD	188.4	11347143
VAST6958665	ViTT	D0	nTD	142.7	17503301

VAST6958666	ViTT	D0	TD	154	12301805
VAST6958667	Control	D0	TD	78.5	16125899
VAST6958668	ViTT	D0+12h	nTD	110	14343800
VAST6958669	ViTT	D0+12h	TD	147	13178447
VAST6958670	ViTT	D0+12h	nTD	199.5	18382938
VAST6958671	ViPS	D0	nTD	255.5	13836877
VAST6958672	ViTT	D0	TD	257.5	13550451
VAST6958673	ViTT	D0	nTD	101	15196159
VAST6958675	ViTT	D0	nTD	115	15898564
VAST6958676	ViPS	V7	nTD	19.5	17972159
VAST6958677	ViPS	V0	nTD	206.8	21534734
VAST6958678	ViPS	V1	TD	275.5	17413468
VAST6958679	ViPS	V1	nTD	111.7	19626923
VAST6958680	ViTT	D0	TD	154.3	14859972
VAST6958681	ViTT	D0	nTD	166.5	12831562
VAST6958682	ViTT	D7	nTD	92.1	15759865
VAST6958683	Control	D0	TD	290.8	15326549
VAST6958684	ViPS	D0	nTD	72.5	17037828
VAST6958685	ViPS	V0	TD	107.3	18008621
VAST6958686	ViTT	D7	nTD	120	18915994
VAST6958687	ViTT	D7	nTD	106.7	15064925
VAST6958688	ViPS	V7	TD	113.2	14599811
VAST6958689	ViTT	D7	nTD	53.7	17459348
VAST6958690	ViPS	V7	nTD	51.3	18897855
VAST6958691	ViTT	D0	nTD	131.1	13624986
VAST6958692	ViTT	D0	nTD	183.2	13034619
VAST6958693	ViTT	D7	nTD	63.9	16875625
VAST6958694	ViTT	D0	nTD	69.5	15181807
VAST6958695	ViTT	TD	TD	74.2	13153394
VAST6958696	ViTT	D0	TD	123.8	14586113
VAST6958697	ViTT	D0	nTD	121.4	15511987
VAST6958698	ViPS	V1	TD	58.2	16252323
VAST6958699	ViTT	D0+12h	TD	151	14355241
VAST6958700	ViPS	V0	TD	69.2	15455191
VAST6958701	ViPS	D0+12h	nTD	81.9	19095324
VAST6958702	ViTT	D0+12h	TD	163.6	14095326
VAST6958703	ViPS	V1	TD	63.3	15967559
VAST6958704	ViPS	V7	TD	58.6	13780406
VAST6958705	ViTT	D0+12h	TD	157.4	13111711
VAST6958706	ViPS	V7	TD	93.3	17290062
VAST6958707	ViTT	D0+12h	nTD	158.7	14465293
VAST6958708	ViTT	D0+12h	nTD	131.2	15475465
VAST6958709	Control	D0	TD	123.7	16207698
VAST6958710	Control	D0	TD	122.2	15960817
VAST6958711	Control	D0	nTD	67.8	12212237
VAST6958712	ViPS	D0	nTD	144.9	13156750
VAST6958713	Control	D0	TD	57	18233786
VAST6958714	ViPS	V0	TD	36.1	19282626

VAST6958715	ViPS	V0	nTD	94.2	17336468
VAST6958716	ViPS	V0	nTD	115.9	13390386
VAST6958717	ViPS	V1	TD	139.7	16168957
VAST6958718	ViPS	V0	nTD	117.2	22231839
VAST6958719	ViTT	TD	TD	128.4	13300233
VAST6958720	ViPS	V0	nTD	193.1	12856223
VAST6958721	ViPS	V1	nTD	71.3	16389057
VAST6958722	ViPS	V1	nTD	101.1	16407614
VAST6958723	ViPS	V1	nTD	170.9	15944028
VAST6958724	ViPS	V1	nTD	116.8	19676689
VAST6958725	ViPS	D0	TD	84.3	14721504
VAST6958726	ViPS	D7	nTD	53.1	18326898
VAST6958727	ViTT	TD	TD	80.9	12364291
VAST6958728	ViPS	D0	nTD	56.3	15090826
VAST6958729	ViTT	D7	nTD	195.4	11902224
VAST6958730	ViPS	V7	nTD	69.1	13151618
VAST6958731	ViTT	D7	nTD	135.9	12744357
VAST6958732	Control	D0	nTD	67.4	16495654
VAST6958733	ViPS	V7	TD	80.7	16347254
VAST6958734	ViPS	V7	nTD	34.4	17635374
VAST6958735	ViPS	V7	nTD	100.7	15046643
VAST6958736	ViPS	D0	TD	78	13945462
VAST6958737	ViTT	TD	TD	146	13230190
VAST6958738	Control	D0	TD	99.3	13372433
VAST6958739	ViPS	V0	TD	72.9	17412969
VAST6958740	Control	D0	TD	132.8	13584427
VAST6958741	ViTT	D0+12h	TD	145.5	16317951
VAST6958742	Control	D0	TD	65.9	18735057
VAST6958743	ViTT	D0+12h	nTD	112.3	12680271
VAST6958744	ViPS	D0	TD	79.1	13433979
VAST6958745	ViTT	D0+12h	nTD	154.5	13961714
VAST6958746	Control	D0	nTD	112.2	13390237
VAST6958747	ViTT	D0+12h	nTD	164.2	13808886
VAST6958748	ViPS	V0	nTD	99	16755111
VAST6958749	Control	D0+12h	TD	109.2	13566617
VAST6958750	ViPS	D0	nTD	82	16033973
VAST6958751	ViPS	V1	nTD	122.5	12512998
VAST6958752	ViPS	V1	TD	66.6	10642210
VAST6958753	ViTT	D0+12h	nTD	129	12518255
VAST6958754	ViPS	D0+12h	nTD	122.3	13753767
VAST6958755	ViPS	V7	nTD	129	14029624
VAST6958756	ViPS	V7	TD	124.3	12872385
VAST6958757	ViPS	D0	TD	91.7	12303101
VAST6958758	ViPS	V0	nTD	66.9	14767451
VAST6958759	Control	D0	TD	85.5	16226770
VAST6958760	ViPS	D0	nTD	77.1	15425140
VAST6958761	ViPS	D0	nTD	111.7	19366293
VAST6958762	Control	D0	TD	61.5	21848905

VAST6958764	Control	D0	TD	110.9	15388724
VAST6958765	ViPS	V0	TD	78.8	15679280
VAST6958766	ViPS	V0	TD	78.4	16397390
VAST6958767	ViPS	V1	nTD	111.5	14925120
VAST6958768	ViPS	V1	TD	95.2	17632270
VAST6958769	ViPS	V0	TD	43.9	16115681
VAST6958770	ViPS	V7	nTD	81	15647767
VAST6958771	ViPS	V0	TD	78.7	16893984
VAST6958772	ViPS	V1	TD	132.1	17690308
VAST6958773	Control	TD	TD	115.4	19075330
VAST6958774	ViPS	D0	TD	92.9	18455285
VAST6958775	ViPS	D0	nTD	101.5	17936226
VAST6958776	Control	D0	nTD	78.8	15456835
VAST6958777	ViPS	V7	TD	159.6	16692658
VAST6958778	ViTT	D7	nTD	180.8	18625814
VAST6958779	ViTT	D7	nTD	155.7	16146116
VAST6958780	ViPS	V0	nTD	35.7	21152638
VAST6958781	ViTT	D7	nTD	182.5	13516060
VAST6958782	ViPS	V0	nTD	50.5	20864851
VAST6958783	ViPS	V1	TD	73.4	14404438
VAST6958784	ViPS	V1	nTD	88.9	16819121
VAST6958785	ViTT	TD	TD	103.9	16683094
VAST6958786	ViTT	D7	nTD	120.7	16867713
VAST6958787	ViPS	D0	TD	157.2	16763361
VAST6958788	Control	D0+12h	TD	128.8	16017487
VAST6958789	ViPS	D7	nTD	109.4	14624602
VAST6958790	ViPS	V1	nTD	45.2	16429057
VAST6958791	ViPS	V7	nTD	136.9	13943742
VAST6958792	ViTT	TD	TD	155.6	15741824
VAST6958793	ViPS	V7	TD	102.4	17160897
VAST6958794	ViPS	D0	nTD	126.5	16330465
VAST6958795	Control	D0	TD	99.3	11730684
VAST6958796	ViTT	D0+12h	TD	134.3	14745018
VAST6958797	ViPS	D0	TD	74.4	20400137
VAST6958798	ViPS	D0+12h	nTD	125.7	18829178
VAST6958799	Control	D0	TD	150.9	13504233
VAST6958800	ViTT	D0+12h	nTD	136.4	12434429
VAST6958801	ViPS	D0	TD	136.9	15553634
VAST6958802	ViTT	D0+12h	TD	173.8	15115717
VAST6958803	ViPS	V0	TD	110.1	15390035
VAST6958804	ViPS	V7	nTD	70.3	16715393
VAST6958805	ViPS	V1	TD	99.3	17405998
VAST6958806	ViPS	V7	TD	85.3	18956504
VAST6958807	ViTT	D0+12h	nTD	180.1	11151761
VAST6958808	ViTT	D0+12h	nTD	166.9	10899744
VAST6958809	ViTT	D0+12h	nTD	135.9	14822095
VAST6958810	ViTT	D0+12h	nTD	110.4	12246684
VAST6958811	Control	D0	TD	106.8	16506361

VAST6958812	ViTT	D0+12h	nTD	122.1	14454443
VAST6958813	Control	D0	TD	144.6	15537923
VAST6958814	ViPS	D0	nTD	55	17236716
VAST6958815	ViPS	D0	nTD	118.7	15046954
VAST6958816	ViPS	D0	TD	67	13096968
VAST6958817	Control	D0	TD	103.6	11596645
VAST6958819	ViPS	D7	nTD	76.7	17149328
VAST6958820	Control	TD	TD	123.3	15954193
VAST6958821	ViTT	D7	nTD	204.7	14207042
VAST6958822	ViTT	D7	nTD	130.6	14888085
VAST6958823	ViTT	D7	nTD	235.2	10788081
VAST6958824	ViTT	TD	TD	163.2	12509251
VAST6958825	ViTT	D7	nTD	143.8	11529288
VAST6958826	ViTT	TD	TD	192.4	13593892
VAST6958827	ViTT	D14	nTD	81.3	15785771
VAST6958828	Control	D0+12h	nTD	111	17050813
VAST6958829	Control	D0+12h	TD	95.7	18684948
VAST6958830	Control	D0+12h	TD	97.1	17563083
VAST6958831	ViPS	D0+12h	nTD	133	13009044
VAST6958832	Control	D0+12h	TD	148.6	12682584
VAST6958833	ViTT	D7	nTD	68.5	12566508
VAST6958834	Control	TD	TD	54.9	11458836
VAST6958835	Control	TD	TD	72.8	11960841
VAST6958836	ViPS	D0+12h	nTD	73.2	14230585
VAST6958837	ViPS	D0+12h	TD	164.8	16344365
VAST6958838	Control	D7	nTD	101.8	18222782
VAST6958839	Control	TD	TD	68.9	12584820
VAST6958840	ViPS	D7	nTD	92.8	12595022
VAST6958841	Control	D0+12h	nTD	240.8	10523762
VAST6958842	ViPS	D7	nTD	59.7	12309550
VAST6958843	Control	D0+12h	TD	111.1	13211015
VAST6958844	Control	D0+12h	nTD	133.5	16875026
VAST6958845	Control	D0+12h	TD	92.3	12907296
VAST6958846	ViPS	D0+12h	TD	98.6	16768065
VAST6958847	ViPS	TD	TD	127.7	12017858
VAST6958848	Control	D0+12h	TD	57.4	11550789
VAST6958849	ViPS	D0+12h	TD	135.1	11923981
VAST6958850	ViPS	TD	TD	63.1	13686619
VAST6958851	Control	TD	TD	78.8	11456982
VAST6958852	Control	D7	nTD	81.9	12499242
VAST6958853	ViPS	TD	TD	114.7	13379052
VAST6958854	Control	TD	TD	104.6	13331480
VAST6958855	Control	D7	nTD	167	16963309
VAST6958856	ViPS	D0+12h	nTD	122	15007341
VAST6958857	Control	TD	TD	47.9	21347861
VAST6958858	Control	D0+12h	TD	86.9	20139209
VAST6958859	ViPS	D0+12h	nTD	200.6	18532355
VAST6958860	ViPS	D0+12h	nTD	55.9	19280125

VAST6958861	ViPS	D0+12h	TD	83.5	14800360
VAST6958862	ViPS	D0+12h	nTD	106.9	19173613
VAST6958863	Control	D0+12h	TD	89.7	16048672
VAST6958864	Control	D0+12h	TD	104.9	19471471
VAST6958865	Control	TD	TD	106.5	16894204
VAST6958866	Control	TD	TD	85.9	17508492
VAST6958867	Control	TD	TD	69.8	20191103
VAST6958868	Control	TD	TD	68.7	24022536
VAST6958869	ViPS	D7	nTD	170.6	19899732
rVAST695887	Control	D0+12h	nTD	136.8	22735442
rVAST695887	ViPS	D7	nTD	109.6	17741244
VAST6958872	ViPS	D0+12h	TD	104.3	20669313
VAST6958873	Control	D7	nTD	107.7	19516705
VAST6958874	ViPS	TD	TD	117.3	20988416
VAST6958875	ViPS	D7	nTD	90.4	22640647
VAST6958876	ViPS	D0+12h	TD	131.8	22762013
VAST6958877	Control	D0+12h	TD	112.1	20903176
VAST6958878	ViPS	D0+12h	nTD	140.5	18890654
VAST6958879	Control	D0+12h	TD	137	16270016
VAST6958880	ViPS	D0+12h	TD	123.5	18706079
VAST6958881	Control	TD	TD	75.5	18300161
VAST6958882	ViPS	TD	TD	121.1	20245619
VAST6958883	ViPS	D7	nTD	128.6	18199134
VAST6958884	Control	TD	TD	128.9	15446048
VAST6958885	ViPS	D0+12h	nTD	111.6	18485140
VAST6958886	ViPS	D0+12h	TD	101.8	20281110
VAST6958887	ViPS	D0+12h	nTD	59.7	18256133
VAST6958888	Control	D0+12h	TD	162.3	18819205
VAST6958889	Control	D0+12h	TD	138.5	17788700
VAST6958890	Control	TD	TD	53.1	18867196
VAST6958891	ViPS	TD	TD	84.9	16487096
VAST6958892	ViPS	D7	nTD	53	17384188
VAST6958893	ViPS	D7	nTD	115.6	15784583
VAST6958894	ViPS	D0+12h	TD	128.3	16078883
VAST6958895	ViPS	TD	TD	68.8	19284172
VAST6958896	Control	D0+12h	TD	128.7	18038033
VAST6958897	Control	TD	TD	91.1	15490996
